# Corollary Discharge Dysfunction to Inner Speech and its Relationship to Auditory Verbal Hallucinations in Patients with Schizophrenia Spectrum Disorders

**DOI:** 10.1093/schbul/sbaf167

**Published:** 2025-10-21

**Authors:** Thomas J Whitford, Lawrence Kin-hei Chung, Oren Griffiths, Bradley N Jack, Mike E Le Pelley, Kevin M Spencer, Ana R Barreiros, Anthony W Harrison, Nathan T Han, Sol Libesman, Daniel Pearson, Ruth B Elijah, Marianthe Godwin, Carla Haroutonian, Angela Nickerson, Sandra Sau-man Chan, George Heung-chuen Chong, Gary Kar-wai Lau, Yip-chau Wong, Jason Wai-yiu Wong, Judith M Ford, Daniel H Mathalon, Anthony W F Harris, Suzanne Ho-wai So

**Affiliations:** School of Psychology, University of New South Wales (UNSW Sydney), Sydney, 2052, Australia; Brain Dynamics Centre, Westmead Institute for Medical Research, Sydney, 2145, Australia; School of Psychology, University of New South Wales (UNSW Sydney), Sydney, 2052, Australia; Department of Psychology, The Chinese University of Hong Kong, Hong Kong SAR, 999077, China; School of Psychological Sciences, The University of Newcastle, Callaghan, 2308, Australia; Research School of Psychology, Australian National University, Canberra, 2600, Australia; School of Psychology, University of New South Wales (UNSW Sydney), Sydney, 2052, Australia; Veterans Affairs Boston Healthcare System, Boston, MA, 02130-4817, United States; Department of Psychiatry, Harvard Medical School, Boston, MA, 02115, United States; Brain Dynamics Centre, Westmead Institute for Medical Research, Sydney, 2145, Australia; School of Psychology, The University of Sydney, Sydney, 2006, Australia; School of Psychology, University of New South Wales (UNSW Sydney), Sydney, 2052, Australia; Department of Psychology and Behavioural Sciences, Zhejiang University, Hangzhou, 310058, China; NHMRC Clinical Trial Centre, The University of Sydney, Sydney, 2006, Australia; School of Psychology, The University of Sydney, Sydney, 2006, Australia; School of Psychology, University of New South Wales (UNSW Sydney), Sydney, 2052, Australia; School of Psychology, University of New South Wales (UNSW Sydney), Sydney, 2052, Australia; Brain Dynamics Centre, Westmead Institute for Medical Research, Sydney, 2145, Australia; Faculty of Medicine and Health Sciences, The University of Sydney, Sydney 2006, Australia; School of Psychology, University of New South Wales (UNSW Sydney), Sydney, 2052, Australia; Department of Psychiatry, The Chinese University of Hong Kong, Hong Kong SAR, 999077, China; Clinical Psychology Service, Kwai Chung Hospital, Hong Kong SAR, 999077, China; Department of Psychiatry, Tai Po Hospital, Hong Kong SAR, 999077, China; Department of Psychiatry, Tai Po Hospital, Hong Kong SAR, 999077, China; Department of Psychiatry, Prince of Wales Hospital, Hong Kong SAR, 999077, China; Department of Psychiatry, North District Hospital, Hong Kong SAR, 999077, China; Department of Psychiatry, University of California – San Francisco, CA, 94143, United States; Department of Psychiatry, University of California – San Francisco, CA, 94143, United States; Brain Dynamics Centre, Westmead Institute for Medical Research, Sydney, 2145, Australia; Discipline of Psychiatry, The University of Sydney, Sydney, 2006, Australia; Department of Psychology, The Chinese University of Hong Kong, Hong Kong SAR, 999077, China

**Keywords:** schizophrenia, corollary discharge, covert speech, speaking-induced suppression, event-related potential, N1, efference copy

## Abstract

**Background and Hypothesis:**

Auditory-verbal hallucinations (AVH)—the experience of hearing voices in the absence of auditory stimulation—are a cardinal psychotic feature of schizophrenia-spectrum disorders. It has long been suggested that some AVH may reflect the misperception of inner speech as external voices due to a failure of corollary-discharge-related mechanisms. We aimed to test this hypothesis with an electrophysiological marker of inner speech.

**Study Design:**

Participants produced an inner syllable at a precisely specified time, when an audible syllable was concurrently presented. The inner syllable either matched or mismatched the content of the audible syllable. In the passive condition, participants did not produce an inner syllable. We compared the amplitude of the N1, P2, and P3-components of the auditory-evoked potential between: (1) schizophrenia-spectrum patients with current AVH (SZAVH+, *n* = 55), (2) schizophrenia-spectrum patients without current AVH (SZAVH−, *n* = 44), (3) healthy controls (HC, *n* = 43).

**Study Results:**

The HC group showed reduced N1-amplitude in the Match condition (relative to Passive and Mismatch), replicating our previous results. In contrast, the SZAVH+ group showed the opposite effect: *enhanced* N1-amplitude in the Match condition (relative to Passive and Mismatch). The SZAVH− group showed reductions in the Mismatch condition (relative to Passive and Match).

**Conclusions:**

This study provides empirical support for the theory that AVH are related to abnormalities in the normative suppressive mechanisms associated with inner speech. This phenomenon of “inner speaking-induced suppression” may have utility as a biomarker for schizophrenia-spectrum disorders generally, and may index a tendency for AVH specifically at more extreme levels of abnormality.

## Introduction

Auditory-verbal hallucinations (AVH), a cardinal feature of schizophrenia-spectrum disorders, are characterized by the perception of voices without corresponding external stimulation. AVH have long been posited to reflect self-generated thoughts being misperceived as external voices.^1–5^ This abnormality may be caused by failures of the corollary discharge mechanisms which typically use a copy of the motor signal to predict, and often suppress, the sensory consequences of self-generated actions, and potentially also self-generated thoughts, as per Hughlings Jackson’s conceptualization of thoughts as our “most complex motor act.”^6^ An abnormality in these mechanisms may obscure the distinction between “self,” and “world,” and hence lead to the misattribution of self-generated thoughts and actions to external sources.^1^^,^^3^^,^^7–9^

Electroencephalography (EEG) studies have consistently shown that healthy individuals exhibit speaking-induced suppression (SIS), which is the phenomenon that self-generated speech elicits a smaller neurophysiological response in the auditory cortex than the same sounds externally-generated. SIS has commonly been operationalized as a reduction in the amplitude of the N1-component of the auditory-evoked potential to self-generated speech sounds.^10–14^ Patients with schizophrenia have consistently been found to exhibit subnormal levels of SIS, that is, they show lower levels of N1-suppression to their own speech sounds compared to matched healthy controls^15–19^—see^20^ for a review. These SIS deficits in schizophrenia have been argued to reflect a failure to suppress the activity of the auditory cortex to self-generated speech due to a corollary-discharge-related dysfunction.

However, while this evidence suggests that schizophrenia patients show abnormalities in the normative suppression of *overt* speech, the central tenet of the corollary discharge theory of AVH suggests that AVH arises from the misperception of *inner* speech—the silent production of words in one’s mind—as external speech.^1^^,^^2^^,^^21–24^ This idea has been difficult to test empirically because of the inherently covert nature of inner speech. To this end, we have recently developed an experimental protocol which aims to investigate corollary discharge function in the context of inner speech.^25^^,^^26^ In this protocol, we asked healthy participants to imagine a single syllable (eg, inner syllable /ba/) that could either match (eg, auditory probe /ba/) or mismatch (eg, auditory probe /bi/) the content of a time-locked auditory probe. Critically, we found that production of inner speech resulted in suppression of the N1 elicited by the auditory probe (relative to a passive listening condition), but only if the content of the inner speech matched the content of the auditory probe.^25^^,^^27^ Furthermore, if the auditory probe was time-shifted by a few hundred milliseconds before or after the inner syllable, no N1-suppression was observed.^26^ Taken together, these results suggest that N1-suppression to inner speech may reflect an objective marker of inner speech that is sensitive to both its content and temporal onset. This provides a potential framework for exploring the long-posited but difficult-to-test hypothesis that abnormalities in inner speech underlie AVH in schizophrenia.

The present study aimed to investigate abnormalities of N1-suppression to inner speech in patients with schizophrenia spectrum disorders by employing the same paradigm as Whitford et al.^25^ While N1-suppression deficits to overt speech in schizophrenia have been well-established, a clear demonstration of N1-suppression deficits to inner speech is still lacking. Given the strong theoretical association between inner speech abnormalities and AVH, the present study adopted a 3-group design; namely, we compared individuals with a schizophrenia spectrum disorder with current AVH (SZAVH+), individuals with a schizophrenia spectrum disorder without current AVH (SZAVH−), and healthy controls (HC). We hypothesized that while the HC and SZAVH− participants would show the typical pattern of “inner speaking-induced suppression” (ie, N1-suppression to inner speech, but only when the inner and audible syllables were matched on content), the SZAVH+ participants would not exhibit this “inner SIS” effect, given the hypothesized association between “inner SIS” deficits and AVH.

## Methods

### Participants

Three groups of adult participants were recruited for the study: SZAVH+, SZAVH−, and HC. In order to augment the participant sample size and increase the statistical power, recruitment and testing was conducted at 2 separate sites, namely the Westmead Institute for Medical Research, Westmead Hospital, Sydney, Australia, and the Department of Psychology, The Chinese University of Hong Kong, Hong Kong—see Table 1 for a breakdown of the participant sample by group and testing site. While the experimental protocol and EEG data analysis protocol were identical across the 2 testing sites, the EEG hardware differed between the 2 sites. While this was not anticipated to be problematic given the within-subjects nature of the key dependent variable (N1-suppression), testing site was nonetheless included as a nuisance covariate in the statistical analysis (see section “Statistical Analysis”).

**Table 1:** Demographic and Clinical Information for the Study Participants

	**Hallucinators (SZAVH+)**	**Non-hallucinators (SZAVH−)**	**Healthy controls (HC)**	**Between-group comparison (*P*-value)**
Number of participants	55	44	43	
Age (years)	42.35 (12.72)	39.91 (10.84)	43.53 (13.21)	.375
Gender	27F, 28M	19F, 25M	21F, 22M	.815
Education	5P, 24S, 17D/T, 4B, 1M, 4NA	1P, 23S, 11D/T, 9B	1P, 9S, 3D/T, 17B, 8M, 5NA	<.001^*^
Employment	23Y, 28N, 4NA	14Y, 28N, 2NA	31Y, 7N, 5NA	<.001^*^
Diagnosis (SCID)	48SCZ, 5SAD, 1SFD, 1PD-NOS	33SCZ, 5SAD, 4DD, 2BPD		.085
Past history of AVH	55Y	28Y, 15N, 1NA		<.001^*^
History of hospitalization	33Y, 9N, 13NA	28Y, 11N, 5NA		.480
Antipsychotic medication class	3U, 43A, 5A + T, 4NA	4U, 36A, 2T, 2NA		.121
Olanzapine equivalent (mg/day)	18.28 (11.53)^a^	12.65 (10.30)^b^		.024^*^
PANSS P3	4.22 (0.94)	1.20 (0.59)		<.001^*^
PANSS positive	18.55 (5.50)	12.77 (5.02)		<.001^*^
PANSS negative	12.87 (5.42)	16.80 (8.39)		.009^*^
PANSS general psychopathology	28.13 (7.42)	28.34 (9.51)		.900
PANSS total	59.55 (15.91)	57.91 (19.97)		.651
PSYRATS AH	23.19 (7.01)	0 (0)		<.001^*^
PSYRATS D	11.91 (7.04)^c^	5.41 (7.12)^d^		<.001^*^
PSYRATS total	35.27 (11.94)^c^	5.41 (7.12)^d^		<.001^*^
Handedness	5L, 42R, 2A, 6NA	1L, 41R, 1A, 1NA	36R, 2A, 5NA	.177
Location	22AU, 33HK	15AU, 29HK	14AU, 29HK	.714

Mean and standard deviation are presented where appropriate. Education: P, primary school; S, secondary school; D/T, diploma or trade school; B, bachelor’s degree; M, master’s degree; NA, not available. Employment, Past history of AVH, History of hospitalization: Y, yes; N, no; NA, not available. Diagnosis: SCZ, schizophrenia; SAD, schizoaffective disorder; DD, delusional disorder; SFD, schizophreniform disorder, BPD, brief psychotic disorder; PD-NOS, psychotic disorder NOS. Antipsychotic medication class: U, unmedicated; A, atypical antipsychotic; T, typical antipsychotic; NA, not available. PANSS, Positive and Negative Syndrome Scale. PSYRATS, Psychotic Symptom Rating Scales. Handedness: L, left; R, right; A, ambidextrous; NA, not available. Location: AU, Australia; HK, Hong Kong. Chi-squared, *t*-test, and ANOVAs were used as appropriate, and statistically significant comparisons (*P* < .05) are indicated with asterisks. ^a^*n* = 45. ^b^*n* = 37. ^c^*n* = 53. ^d^*n* = 42.

For the SZAVH+ and SZAVH− participants, inclusion criteria were a diagnosis of a schizophrenia spectrum disorder (schizophrenia, schizoaffective disorder, delusional disorder; schizophreniform disorder, brief psychotic disorder; psychotic disorder not otherwise specified) based on the Structured Clinical Interview for DSM-IV Axis I Disorders (SCID-I/P),^28^ and an age of at least 18 years. The presence of AVH was defined by scoring 3 or above on item P3 (hallucinatory behavior) of the Positive and Negative Syndrome Scales (PANSS),^29^ with follow-up on the Psychotic Symptoms Rating Scales (PSYRATS)^30^ indicating that the hallucinatory experiences were auditory-verbal in nature. Most participants in the SZAVH− group received a score of 2 or less on the P3 item of the PANSS (the minimum possible score on this item was 1); 2 participants in this group received a P3 score of 3, however, follow-up revealed that their hallucinations did not occur in the auditory modality. HC participants without a present or past diagnosis of an Axis I disorder (based on SCID-I/P) were included, and were age- and gender-matched to the clinical groups as best as possible. All interviews were conducted by a trained psychiatrist, clinical psychologist, or research assistant.

SZAVH+ and SZAVH− participants were referred by clinicians at Westmead Hospital, Sydney, and public hospitals in the New Territories East Cluster and Kowloon West Cluster, Hong Kong. HC participants were recruited by advertisements and word-of-mouth. Exclusion criteria for all groups included a history of alcohol or substance dependence within the past year (except nicotine dependence), a history of a significant medical or neurological illness, drug-induced or organic psychosis, and intellectual disability.

In total, 58 SZAVH+, 51 SZAVH−, and 49 HC were recruited for the study. Fifteen participants were excluded from the analysis due to excessive artifacts in the EEG recording, defined as generating ≤25% of usable epochs in one or more conditions, and one participant was excluded as their nose electrode was not successfully recorded. The final sample consisted of 55 SZAVH+, 44 SZAVH−, and 43 HC participants. All participants self-reported to have normal or corrected-to-normal vision and hearing. The study was approved by the Western Sydney Local Health District Human Research Ethics Committee, the Joint Chinese University of Hong Kong—New Territories East Cluster Clinical Research Ethics Committee, and the Kowloon West Cluster Research Ethics Committee, and conducted in accordance with the Declaration of Helsinki. All participants provided written informed consent.

The demographic and clinical details of the study participants are summarized in Table 1. Symptom severity ratings were obtained from SZAVH+ and SZAVH− participants by trained raters, using semi-structured interviews for the PANSS^29^ and PSYRATS.^30^ Patients’ medication dosages were converted into olanzapine-equivalents for the purpose of between-group comparison.^31^ Handedness^32^ was also assessed in all participants.

### Inner Speech Paradigm

Participants completed our inner speech paradigm, summarized below, which has been described in detail previously.^25–27^^,^^33^ On each experimental trial, participants viewed a countdown animation on a computer monitor: the ticker tape moved at a constant velocity of 6.5°/s (where ° refers to the visual angle), such that after 3.75 s the trigger line intersected the fixation line (ie, the sound-time) and continued to move beyond (see Figure 1). At the sound-time, an audible syllable /ba/ or a /bi/ would be delivered to participants’ headphones (Sennheiser HD201 in Australia and Focusrite Scarlett HP60 MKII in Hong Kong), which was produced by a male speaker, about 200 ms in duration and about 70 dB SPL in intensity. Each audible syllable (ie, /ba/ and /bi/) was presented on 50% of trials within each trial block in a randomized order. In the active blocks, participants were asked to simultaneously produce an inner syllable (either /ba/ or /bi/), at the sound-time, by imagining themselves moving their articulator organs (ie, mouth, tongue, larynx, etc.) without actually making any movements. They were always asked to imagine the same inner syllable throughout a trial block. In the passive blocks, participants were not required to imagine anything in particular, but were asked to simply listen to the audible syllable (see Figure 1D). The order of the trial blocks was randomized, and there were 24 trials in each trial block. Stimulus presentation was controlled by MATLAB (MathWorks), using the Psychophysics Toolbox extensions.^34^^,^^35^

**Figure 1:**
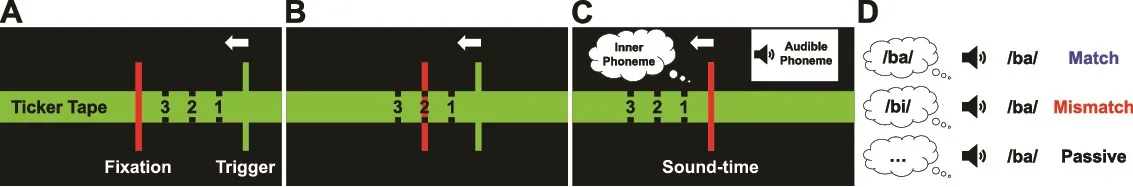
A Schematic of the Experimental Protocol. (A) Participants were asked to fixate their eyes on the stationary fixation line in the middle of the screen. (B) After a delay of 1-2 s, the trigger line began moving leftwards smoothly across the screen from the far right-hand side of the screen at a speed of 6.5°/s. C. After 3.75 s, the trigger line overlapped with the fixation line—a moment dubbed the “sound-time.” At this precise moment, 2 events took place simultaneously. (D) On active trials, participants were instructed to silently produce a pre-defined syllable (either /ba/ or /bi/) in their heads. On passive trials, participants were instructed to passively listen to the audible syllable. At the same time, an audible syllable (either /ba/ or /bi/), produced by a male speaker with a loudness of about 70 dB SPL, was delivered to the participants’ headphones. In the Match trials, the content of the inner syllable was congruent with the audible syllable (eg, inner syllable: /ba/; audible syllable: /ba/). In the Mismatch trials, the content of the inner syllable was incongruent with the audible syllable (e.g., inner syllable: /bi/; audible syllable: /ba/). In the Passive trials, no inner syllable was produced (eg, inner syllable: none; audible syllable: /ba/). Each audible syllable (/ba/ and /bi/) was presented on 50% of trials within each trial block in a randomized order. After the sound-time, the trigger line continued to move past the fixation line for an additional 1 s and stopped. The trial was then completed.

After each trial, participants were asked to rate their subjective performance for that trial on a 5-point Likert scale. Specifically, participants were given the following instructions for the “imagine ba” trials: “After each trial, you will be asked to rate how well you were able to imagine yourself producing the sound ‘ba’ on a scale of 1 (didn’t imagine ‘ba’ at all) to 5 (imagined ‘ba’ perfectly).” The instructions were identical for the “imagine bi” trials, except that “bi” replaced “ba.” For the passive trials, participants were asked: “After each trial, you will be asked to rate how well you were able to listen to the sound, on a scale of 1 (didn’t listen to it at all) to 5 (listened to it perfectly).” In the Match condition, there was an average performance score of 4.49 (SD = 0.62) in SZAVH+, 4.65 (SD = 0.51) in SZAVH−, and 4.83 (SD = 0.24) in HC. In the Mismatch condition, there was an average performance score of 4.17 (SD = 0.88) in SZAVH+, 4.33 (SD = 0.88) in SZAVH−, and 4.39 (SD = 0.87) in HC. In the Passive condition, there was an average performance score of 4.42 (SD = 0.66) in SZAVH+, 4.67 (SD = 0.52) in SZAVH−, and 4.50 (SD = 1.05) in HC. There was a significant difference between the SZAVH+ and HC groups on the trial ratings for the Match condition, controlling for Location (*t*(96) = 3.16, *P* = .002, *d* = 0.32, 95% CI, 0.123-0.540), with the SZAVH+ group rating their performance as lower than the HC group. Similarly, there was a significant difference between the SZAVH+ and SZAVH− groups on the trial ratings for the Passive condition (*t*(97) = 2.02, *P* = .045, *d* = 0.21, 95% CI, 0.007-0.660), with the SZAVH+ group rating their performance as lower than the SZAVH− group. All other contrasts were non-significant.

### E‌EG Acquisition and Preprocessing

Electroencephalographic data were acquired from 64 Ag/AgCl electrodes positioned according to the extended 10-20 system using a Biosemi ActiveTwo system (www.biosemi.com), referenced online to CMS/DRL for participants in Australia. A 64-channel ANT EEGO system (www.ant-neuro.com/), referenced online to CPz was used for participants in Hong Kong. Electro-oculogram data were measured by placing electrodes on the outer canthi of both eyes and above and below the left eye, reflecting horizontal and vertical eye movements, respectively, as well as blinks. Additional electrodes were placed on the bridge of the nose above the face mask (consistent with local COVID-19 masking policies), and on the left and right mastoid. The sampling rate was 2048 Hz for both systems.

The data were pre-processed and analyzed using BrainVision Analyzer (Brain Products GmbH). The EEG data were re-referenced offline to the nose electrode. Data were first notch filtered (50 Hz), and then band-pass filtered from 0.1 to 30 Hz using a phase-shift free Butterworth filter (12 dB/Oct slope). Epochs were extracted from −100 to 400 ms, relative to the audible syllable onset, for 3 separate trial-types: *Match* trials (in which the content of the inner syllable matched the content of the audible syllable; ie, inner syllable /ba/, audible syllable /ba/; or inner /bi/, audible /bi/), *Mismatch* trials (in which the content of the inner syllable mismatched the content of the audible syllable; ie,, inner /ba/, audible /bi/; or inner /bi/, audible /ba/), and *Passive* trials (in which participants were not instructed to produce an inner syllable, ie, inner nil, audible /ba/; or inner nil, audible /bi/).

The segmented epochs were then baseline-corrected to the mean voltage from −100 to 0 ms. All epochs were corrected for eye-movement artifacts, using the technique described in Gratton et al^36^, and any epochs with signals exceeding peak-to-peak amplitudes of 200 μV for any channel were excluded. In the Match condition, there were an average of 82.78 (SD = 20.16) useable epochs in SZAVH+, 86.16 (SD = 15.47) useable epochs in SZAVH−, and 92.28 (SD = 16.98) useable epochs in HC. In the Mismatch condition, there were an average of 82.98 (SD = 19.89) useable epochs in SZAVH+, 85.86 (SD = 15.42) useable epochs in SZAVH−, and 92.21 (SD = 16.97) useable epochs in HC. In the Passive condition, there were an average of 84.67 (SD = 19.85) useable epochs in SZAVH+, 85.66 (SD = 14.64) useable epochs in SZAVH−, and 91.33 (SD = 17.62) useable epochs in HC.

The primary dependent variable was the amplitude of the N1-component of the auditory-evoked potential. N1-amplitude was quantified with a peak-picking approach; the N1 peak was identified as the most negative local minimum potential between 25 and 175 ms post-stimulus onset on each participant’s average waveform, as per our established protocol.^14^^,^^25^^,^^27^^,^^37^^,^^38^

While the primary focus of this article was on the N1-component, we also performed supplementary analyses of the P2 and P3 components. As several participants did not exhibit a clear P2 or P3 peak for one or more conditions, we used a time-window based approach to analyze these components, as we have done previously.^25^^,^^27^ The P2 peak was identified at 186 ms on the collapsed localizer waveform, and a time-window of 166-206 ms was used for the P2 analysis. The P3 peak was identified at 277 ms on the collapsed localizer waveform, and a time window of 247-307 ms was used for the P3 analysis.

The ERP components were analyzed by averaging across the 3 midline electrodes at which the components-of-interest were maximal on the collapsed localizer waveform, which was generated by averaging across all Groups and Conditions.^39^ Electrodes FCz, Cz, and Fz were averaged for the N1 analysis, with the N1-component being maximal at FCz. Electrodes Cz, CPz, and FCz were averaged for the P2 analysis, with the P2-component being maximal at Cz. Electrodes CPz, Cz, and Pz were averaged for the P3 analysis, with the P3-component being maximal at CPz.

### Statistical Analysis

The N1-, P2-, and P3-components were each analyzed separately with a mixed ANOVA. For each component, the between-subjects factor was *Group* (3 levels: SZAVH+, SZAVH−, and HC). The within-subjects factor was *Condition* (3 levels: Match, Mismatch and Passive). The between-subjects factor *Location* (2 levels: Australia and Hong Kong) was also entered as a nuisance covariate. A Greenhouse-Geisser correction was used in the case of a violation in the assumption of sphericity. In the case of a significant *Condition × Group* interaction, follow-up ANOVAs (controlling for *Location*) and contrasts were used to unpack the simple effects. We also used a Spearman’s rank-order correlation to investigate the association between the severity of patients’ auditory-hallucinations (as defined by their score on the Auditory Hallucinations subscale of the PSYRATS), and their between-condition difference scores; that is, N1-amplitude in the Match condition minus the Passive condition (N1-suppression), in the Match condition minus the Mismatch condition, and the Passive condition minus the Mismatch condition.

## Results

### N1-component

Figure 2 shows the results of the N1-component analyses for the 3 conditions (Match in blue, Mismatch in red, Passive in black), for the HC group (Figure 2(i)), the SZAVH+ group (Figure 2(ii)) and the SZAVH− group (Figure 2(iii)). In these figures, Subpanel A shows the grand average auditory-evoked potential waveforms for the 3 conditions; Subpanel B shows the N1 voltage maps for the 3 conditions; Subpanel C shows a box-and-whiskers plot of raw N1-amplitude for the 3 conditions; Subpanel D shows a scatterplot of the between-condition differences in N1-amplitude (within-subjects), which constitute the critical contrasts. Figure 2(iv) summarizes the N1-amplitude means (and within-subjects standard error of the mean) for the 3 conditions, for each of the 3 participant groups. Figure 2(v) shows a scatterplot of participants’ level of N1-suppression (calculated as the difference in N1-amplitude between the Match and Passive conditions) plotted against their total score on the PSYRATS Auditory Hallucinations Scale for the SZAVH+ participants (black circles) and the SZAVH− participants (white circles). Figure 2(vi) shows the corresponding correlations between the PSYRATS Auditory Hallucinations Scale and the Mismatch minus Passive difference score.

**Figure 2:**
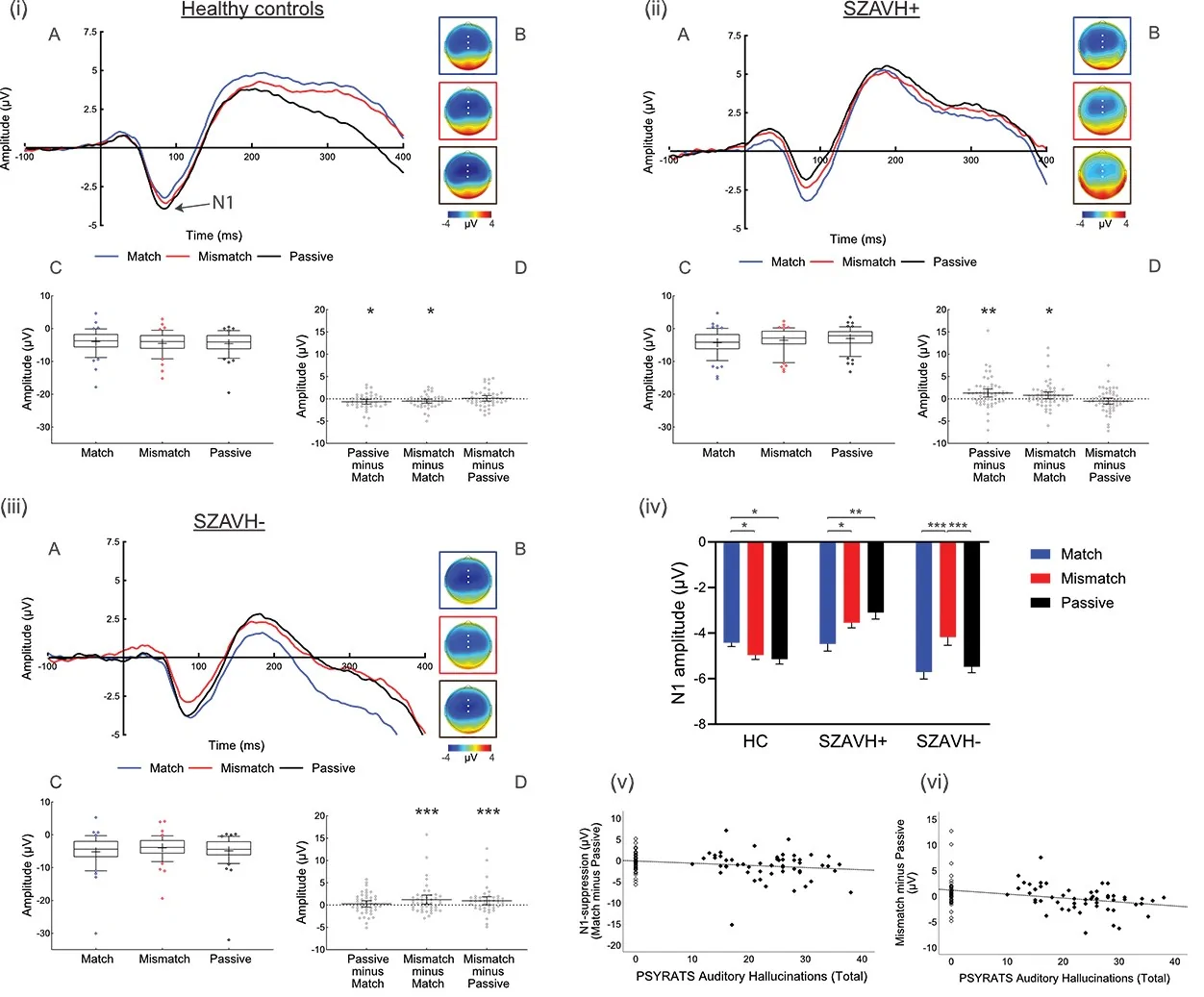
N1-Analysis. Analysis of the auditory-evoked potentials for the 3 conditions (Match, Mismatch, Passive), for the 3 participant groups (HC, SZAVH+, SZAVH−). Panel (i) shows the waveform data for the HC group (*n* = 43), panel (ii) shows the waveform data for the SZAVH+ group (*n* = 55), and panel (iii) shows the waveform data for the SZAVH− group (*n* = 44). Panel (iv) summarizes the N1-amplitude data for the 3 groups (SZAVH+, SZAVH−, HC), for the 3 conditions (Match, Mismatch, Passive). The bars show the estimated marginal-mean (controlling for Location) of the N1-component, while the error-bars show the within-subjects standard error of the mean. Panel (v) shows the correlation between level of N1-suppression (calculated as the difference in N1-amplitude between the Match and Passive conditions) and total score on the PSYRATS Auditory Hallucinations Scale for the participants in the SZAVH+ group (black circles) and the SZAVH− group (white circles). Panel (vi) shows the corresponding correlation for the Mismatch minus Passive difference-score. The dotted line represents the line-of-best-fit. There are 4 subpanels within panels (i)-(iii). Subpanel (A) Waveforms showing the auditory-evoked potentials elicited by the audible syllables averaged across electrodes Fz, FCz, and Cz in the Match condition (blue line), Mismatch condition (red line) and Passive condition (black line). The N1-enhancement effect (i.e., enhanced N1-amplitude in the Match vs. Passive condition) is visible in the SZAVH+ participants in panel (ii), while the N1-suppression effect (ie, suppressed N1-amplitude in the Match vs. Passive condition) is visible in the HC participants in panel (i). The waveforms are shown collapsed across audible syllable (audible syllable /ba/ and /bi/), and the waveforms for the Match and Mismatch conditions are shown collapsed across inner syllable (inner syllable /ba/ and /bi/). Subpanel (B) Voltage maps are plotted separately for each condition; white dots illustrate the electrodes used in the analysis. Subpanel (C) Box-and-whiskers plots showing the amplitude of the N1-component elicited by the audible syllables in the Match, Mismatch, and Passive conditions. The edges of the boxes represent the top and bottom quartiles, the horizontal stripes represent the median, the crosses represent the mean, the whiskers represent the 10th and 90th percentiles, and the colored dots represent the participants whose data fell outside the range defined by the whiskers. Subpanel (D) Scatterplots showing the within-subjects difference scores of N1-amplitude for the contrasts, namely Passive minus Match, Mismatch minus Match, and Mismatch minus Passive. Each dot represents one participant’s difference score. The horizontal bars represent the mean, and the error bars represent the 95% confidence interval. Significant contrasts (*P* < .05) are indicated with asterisks. ^***^P < .001; ^**^P < .01; ^*^P < .05.

The omnibus ANOVA revealed a significant main effect of *Condition*, *F*(2,272) = 3.99, *P* = .020, η_p_^2^ = 0.03 and a significant *Condition × Group* interaction, *F*(4,272) = 6.45, *P* < .001, η_p_^2^ = 0.09. The main effect of *Location* was also significant (*F*(1,136) = 10.001, *P* = .002, η_p_^2^ = 0.07). However, the *Condition × Group × Location* interaction was not statistically significant, *F*(4,272) = 1.37, *P* = .245, η_p_^2^ = 0.02, η_p_^2^ = 0.09 indicating that the strength of the key *Condition × Group* interaction did not significantly differ between the 2 testing locations.

We also investigated the effects of participant age and sex on the above model. There was a main effect of *Age* (*F*(1, 129) = 8.088, *P* = .005, η_p_^2^ = 0.059), with older participants showing a smaller (less negative) N1 response. Similarly, there was also a main effect of *Sex* (*F*(1,129) = 5.745, *P* = .018, η_p_^2^ = 0.043), with female participants showing a larger (more negative) N1 response. Crucially, however, the key *Condition × Group* interaction was preserved when *Age* and *Sex* were both entered as covariates (F(4, 258) = 6.033, *P* < .001, η_p_^2^ = 0.086), when *Age* was entered alone (*F*(4, 270) = 6.618, *P* < .001, η_p_^2^ = 0.089), and when *Sex* was entered alone (*F*(4, 258) = 5.911, *P* < .001, η_p_^2^ = 0.083).

We unpacked the significant *Cognition x Group* interaction by exploring the effect of *Condition* in each of the 3 groups separately. For the HC group, repeated measures ANOVA (controlling for *Location*) revealed a significant main effect of *Condition*, *F*(2, 82) = 3.32, *P* = .041, η_p_^2^ = 0.08. Follow-up contrasts found that N1-amplitude in the *Match* condition was significantly smaller than the *Mismatch* condition, *t*(41) = 2.18, *P* = .035, *d* = 0.33, 95% CI, 0.040-1.036, and the *Passive* condition, *t*(41) = 2.48, *P* = .017, *d* = 0.38, 95% CI, 0.133-1.298. However, the difference between the *Mismatch* and *Passive* conditions was not significant, *t*(41) = 0.54, *P* = .591, *d* = 0.08, 95% CI, −0.484 to 0.838.

For the SZAVH+ group, repeated measures ANOVA (controlling for *Location*) revealed a significant main effect of *Condition*, *F*(2, 106) = 6.42, *P* = .003, η_p_^2^ = 0.11. Follow-up contrasts found that N1-amplitude in the *Match* condition was significantly larger than the *Mismatch* condition, *t*(53) = 2.50, *P* = .016, *d* = 0.34, 95% CI, −1.687 to −0.185, and the *Passive* condition, *t*(53) = 3.03, *P* = .004, *d* = 0.41, 95% CI, −2.293 to −0.466. However, the difference between the *Mismatch* and *Passive* conditions was not significant, *t*(53) = 1.30, *P* = .199, *d* = 0.18, 95% CI, −1.125 to 0.239.

For the SZAVH− group, repeated measures ANOVA (controlling for *Location*) revealed a significant main effect of *Condition*, *F*(2, 84) = 6.69, *P* = .002, η_p_^2^ = 0.14. Follow-up contrasts found that N1-amplitude in the *Mismatch* condition was significantly smaller than the *Match* condition, *t*(42) = 2.97, *P* = .005, *d* = 0.45, 95% CI, 0.491-2.580, and the *Passive* condition, *t*(42) = 2.88, *P* = .006, *d* = 0.43, 95% CI, 0.391-2.215. However, the difference between the *Match* and *Passive* conditions was not significant, *t*(42) = 0.62, *P* = .541, *d* = 0.09, 95% CI, −0.993 to 0.528. Within the SZAVH− group, we also used a repeated-measures ANOVA (controlling for *Location*) to compare those participants who had a past history of AVH (*n* = 28), to those who had no recorded history of AVH (*n* = 15) (*n* = 1 participant had missing data). While the main effect of *Condition* remained significant (*F*(2, 78) = 5.617, *P* = .008, η_p_^2^ = 0.126), there was no main effect of *AVH-History* (*F*(1, 41) = 0.805, *P* = .375, η_p_^2^ = 0.020), and no *Condition x AVH-History* interaction (*F*(2, 78) = 0.084, *P* = .897, η_p_^2^ = 0.002). Finally, there was no difference in N1-suppression score (Match minus Passive) between the 2 groups (*t*(41) = 0.914, *P* = .366, 95% CI, 0.826 to −2.193). Taken together, these results suggest that the variation in N1 amplitude across conditions did not differ significantly between those SZAVH− patients who had a history of AVH and those SZAVH− patients who did not.

With regards to the association between hallucination severity and N1-suppression in the clinical participants: a Spearman’s rank-order correlation identified a significant linear association between clinical participants’ total score on the PSYRATS Auditory Hallucination Scale and their level of N1-suppression (ie, N1-amplitude in the Match condition minus the Passive condition), collapsing across the 3 midline electrodes: rho(99) = −0.222, *P* = .027. The correlation remained statistically significant if the outlier visible at the bottom of Figure 1, panel (v) was removed: rho(98) = −.218, *P* = .031. This correlation also remained statistically significant when controlling for the between-group differences in negative symptom severity (ie, when controlling for PANSS-Negative symptom score using a partial correlation), which provides support for the specificity of this association^40^: rho_partial_(99) = −0.222, *P* = .028. Note that N1-suppression was calculated as Match minus Passive: this means that participants who fell above zero on the y-axis in Figure 1(v) showed “N1- suppression” to the Match condition while participants who fell below zero showed “N1-enhancement” to the Match condition. In other words, participants with higher PSYRATS Auditory Hallucinations score were more likely to show “N1-enhancement.”

We also observed an unpredicted negative correlation between the Mismatch minus Passive difference score and patients’ scores on the PSYRATS Auditory Hallucinations Scale (rho(99) = −0.400, *P* < .001—see Figure 1, panel (vi). This correlation remained significant even when the analysis was limited to the SZAVH+ participants (rho(55) = −0.420, *P* = .001). The correlation between the Match minus Mismatch difference scores and the PSYRATS Auditory Hallucinations Scale was non-significant ((rho(99) = 0.128, *P* = .207).

For completeness, we also conducted Spearman Rank-Order correlations between the 3 N1-difference scores (ie, Match minus Passive, Match minus Mismatch, Mismatch minus Passive) and the clinical participants’ scores on all the other clinical variables, namely PANSS Positive Total, PANSS Negative Total, PANSS General Total, PSYRATS Delusions score, and Olanzapine-Equivalent Medication dosage. These correlations are presented in the Supplementary Material. In short, all of these other correlations were non-significant at *P* < .05.

For completeness, we also performed linear mixed models analyses, following the procedure outlined in Duggirala et al.^41^ The results of these analyses are described in the Supplementary Material section. In these analyses, mean N1 amplitude represented the outcome variable, *Group* (3 levels; HC vs SZAVH+ vs SZAVH−, dummy-coded) was a between-group fixed effect, and *Condition* (3 levels; Passive vs Match vs Mismatch, dummy-coded) was a within-subjects fixed effect. Given data were conducted at 2 sites, we controlled for *Location* (2 levels, Australia vs Hong Kong). A random intercept was included to capture variability in participants’ average N1 amplitude. We investigated whether N1 amplitude varied according to *Group* and *Condition* (over and above *Location*), as described in the above model. Next, we investigated whether the effects of interest were maintained with the inclusion of potential covariates (*Age, Sex*), and whether the associations varied across levels of these covariates (*Age, Sex, Location*). We then investigated whether the association between Condition and Group varied according to the clinical variables (PANSS Negative Total, PANSS General Total, PSYRATS Delusions score, Olanzapine-Equivalent Medication dosage, Age, Sex) in the 2 clinical groups (SZAVH+ and SZAVH−).

### P2-component

Figure 3 shows the results of the P2 component analyses for the 3 conditions (Match in blue, Mismatch in red, Passive in black), for the HC group (Figure 3(i)), the SZAVH+ group (Figure 3(ii)) and the SZAVH− group (Figure 3(iii)). Figure 3(iv) shows the P2-amplitude means (and within-subjects standard error of the mean) for the 3 conditions, for each of the 3 participant groups.

**Figure 3:**
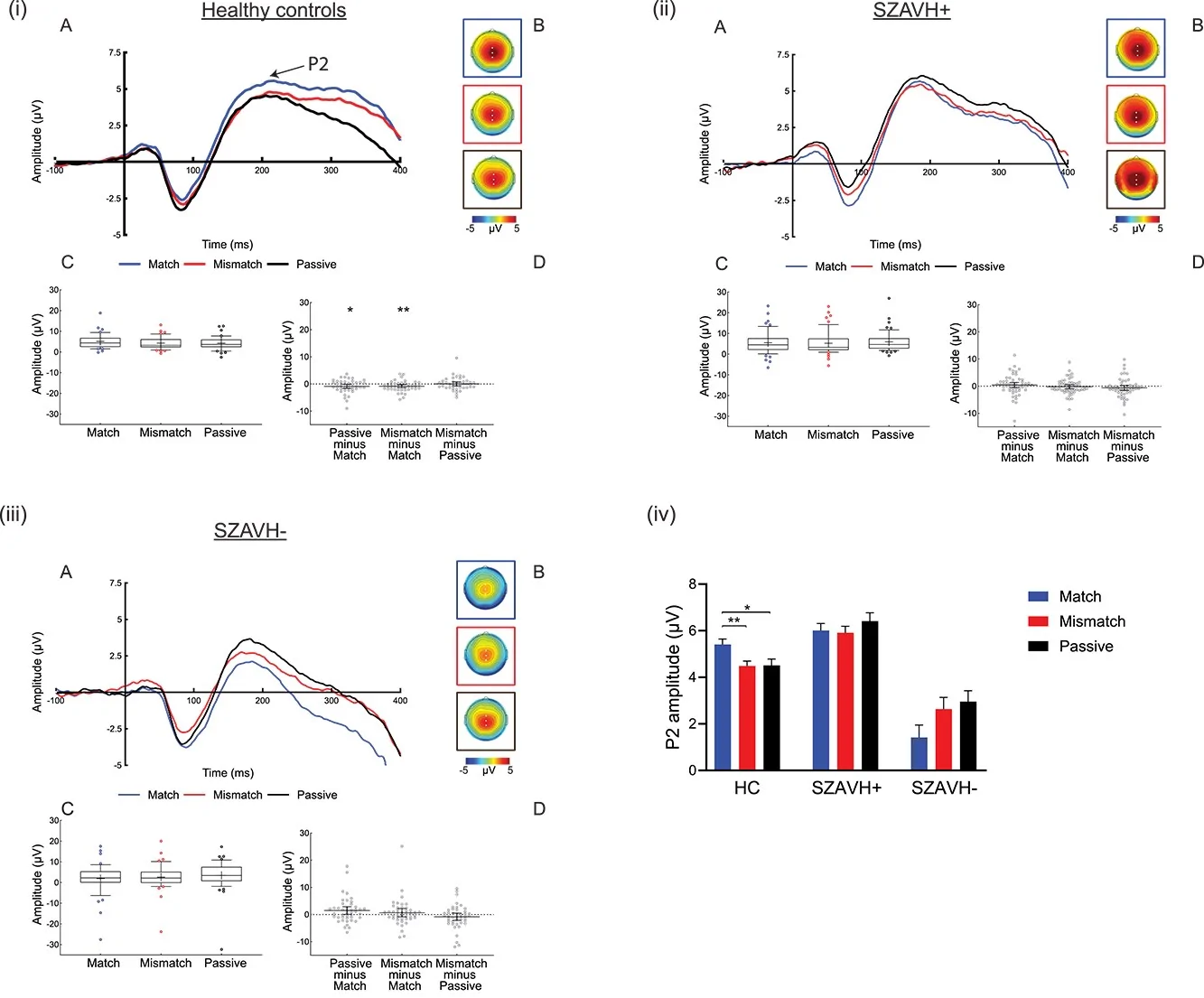
P2-Analysis. Analysis of the P2 component of the auditory-evoked potentials for the 3 conditions (Match, Mismatch, Passive), for the 3 participant groups (HC, SZAVH+, SZAVH−), collapsed across the Cz, CPz, FCz electrodes. Panel (i) shows the waveform data for the HC group (*n* = 43), panel (ii) shows the waveform data for the SZAVH+ group (*n* = 55), and panel (iii) shows the waveform data for the SZAVH− group (*n* = 44). Panel (iv) summarizes the P2-amplitude data for the 3 groups (HC, SZAVH+, SZAVH−), for the 3 conditions (Match, Mismatch, Passive). P2-amplitude was defined as the average voltage in the 166-206 ms time-window. The bars show the estimated marginal-mean (controlling for Location) of the P2-component, while the error-bars show the within-subjects standard error of the mean. The 4 subpanels within panels (i)-(iii) are the same as those already described in Fig. 2: Subpanel (A) is the waveforms, subpanel (B) is the scalp topographies, subpanel (C) is the raw P2 data, subpanel (D) is the P2-difference scores, with significant contrasts highlighted with asterisks. ^***^P < .001; ^**^P < .01; ^*^P < .05.

The omnibus ANOVA revealed a significant *Condition x Group* interaction, *F* (4, 272) = 2.90, *P* = .023, η_p_^2^ = 0.041, and thus the data were broken down into Groups for further analysis.

For the HC group, repeated-measures ANOVA (controlling for *Location*) revealed a main-effect of *Condition*, F(2, 82) = 4.11, *P* =.020, η_p_^2^ = 0.091. Follow-up contrasts revealed that P2-amplitude was significantly higher in the Match condition, relative to both the Mismatch condition (*t*(42) = 2.99, *P* = .005, *d* = 0.46, 95% CI, 0.302-1.561), and the Passive condition (*t*(42) = 2.22, *P* = .032, *d* = 0.34, 95% CI, 0.084-1.719). The Mismatch vs. Passive contrast was non-significant (*t*(42) = 0.08, *P* = .937, *d* = 0.01, 95% CI, −0.745 to 0.806).

For the SZAVH+ group, repeated-measures ANOVA (controlling for *Location*) revealed that the main effect of *Condition* was non-significant (*F*(2 106) = 0.678, *P* = .495, η_p_^2^ = 0.013), and thus follow-up contrasts were not performed.

Similarly, for the SZAVH− group, repeated-measures ANOVA (controlling for *Location*) revealed a non-significant main-effect of *Condition* (*F*(2, 84) = 2.532, *P* = .086, η_p_^2^ = 0.057), and thus follow-up contrasts were not performed.

### P3-component

Figure 4 shows the results of the P3 component analyses for the 3 conditions (Match in blue, Mismatch in red, Passive in black), for the HC group (Figure 4(i)), the SZAVH+ group (Figure 4(ii)), and the SZAVH− group (Figure 4(iii)). Figure 4(iv) shows the P3-amplitude means (and within-subjects standard error of the mean) for the 3 conditions, for each of the 3 participant groups.

**Figure 4:**
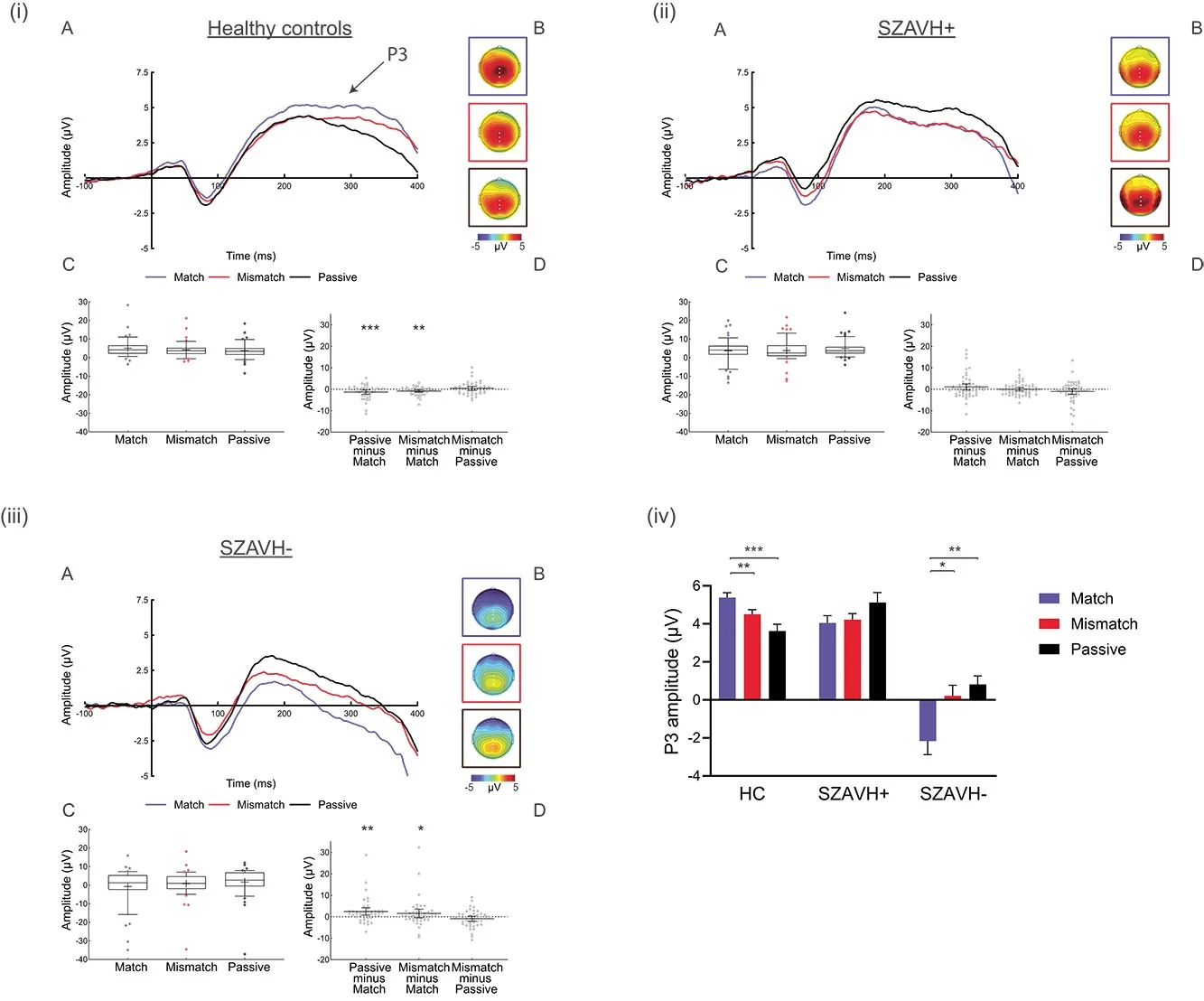
P3 Analysis. Analysis of the P3 component of the auditory-evoked potentials for the 3 conditions (Match, Mismatch, Passive), for the 3 participant groups (HC, SZAVH+, SZAVH−), collapsed across the CPz, Cz, Pz electrodes. Panel (i) shows the waveform data for the HC group (n = 43), panel (ii) shows the waveform data for the SZAVH+ group (*n* = 55), and panel (iii) shows the waveform data for the SZAVH− group (*n* = 44). Panel (iv) summarizes the P3-amplitude data for the 3 groups (HC, SZAVH+, SZAVH−), for the 3 conditions (Match, Mismatch, Passive). P3-amplitude was defined as the average voltage in the 247-307 ms time-window. The bars show the estimated marginal-mean (controlling for Location) of the P3-component, while the error-bars show the within-subjects standard error of the mean. The 4 subpanels within panels (i)-(iii) are the same as those already described in Fig. 2: Subpanel (A) is the waveforms, subpanel (B) is the scalp topographies, subpanel (C) is the raw P3 data, and subpanel (D) is the P3-difference scores, with significant contrasts highlighted with asterisks. ^***^P < .001; ^**^P < .01; ^*^P < .05.

The omnibus ANOVA revealed a significant *Condition × Group* interaction (*F*(4, 272) = 6.742, *P* < .001, η_p_^2^ = 0.09), and thus the data were broken down into Groups for further analysis.

For the HC group, repeated-measures ANOVA (controlling for *Location*) revealed that the main effect of *Condition* was significant (*F*(2,82) = 8.907, *P* < .001, η_p_^2^ = 0.178). Follow-up contrasts revealed that P3-amplitude was significantly higher in the Match condition relative to both the Mismatch condition (*t*(42) = 2.86, *P* = .007, *d* = 0.44, 95% CI, 0.257-1.483), and the Passive condition (*t*(42) = 3.70, *P* < .001, *d* = 0.57, 95% CI, 0.798-2.715). The Mismatch vs. Passive contrast was non-significant (*t*(42) = 1.97, *P* = .055, *d* = 0.30, 95% CI, −1.795 to 0.022).

For the SZAVH+ group, repeated-measures ANOVA (controlling for *Location*) revealed that the main effect of *Condition* was non-significant, (*F*(2, 106) = 1.847, *P* = .174, η_p_^2^ = 0.034), and thus follow-up contrasts were not performed.

For the SZAVH− group, repeated-measures ANOVA (controlling for *Location*) revealed that the main effect of *Condition* was significant (*F*(2, 84) = 6.977, *P* = .003, η_p_^2^ = 0.142). Follow-up contrasts revealed that P3-amplitude was significantly lower in the Match condition relative to both the Mismatch condition (*t*(43) = 2.43, *P* = .020, *d* = 0.37, 95% CI, 0.399-4.345), and the Passive condition (*t*(43) = 3.35, *P* = .002, *d* = 0.51, 95% CI, 1.183-4.763). The Mismatch vs. Passive contrast was not significant (*t*(43) = 0.97, *P* = .337, *d* = 0.15, 95% CI, −0.648 to 1.849).

## Discussion

The key results can be summarized as follows: the healthy control participants (HC) exhibited the expected pattern of “inner speaking-induced suppression” (“inner SIS”); that is, they exhibited reductions in N1-amplitude in the auditory-evoked potential elicited by the audible syllable (relative to the Passive condition), but most strongly when the content of the audible syllable matched the content of the concurrently-produced inner syllable, that is, in the Match condition. In contrast, the 2 groups of schizophrenia-spectrum patients did not exhibit this normal pattern of “inner SIS.” The patients with current auditory-verbal hallucinations (SZAVH+) actually showed an *inversion* of the typical inner SIS effect: that is, they showed an *enhanced* N1-amplitude in the Match condition, relative to both the Passive and Mismatch conditions. The patients without current auditory-verbal hallucinations (SZAVH−) showed a somewhat different pattern: N1-amplitude was reduced in the Mismatch condition relative to both the Passive and Match conditions, which did not differ from each other.

As noted earlier, it has long been argued that some of the most characteristic symptoms of schizophrenia could arise from an abnormality in the neural mechanisms involved in distinguishing between self-generated actions and externally-generated events.^1–5^^,^^42^ Consistent with this theory, schizophrenia patients have been shown to exhibit deficits in predicting and suppressing the sensory consequences of their own physical actions, including eye-movements (eg, saccades),^43^^,^^44^ hand movements,^38^^,^^45^ and overt speech.^15–19^^,^^46^^,^^47^ In a similar vein, it has been suggested that corollary-discharge-related abnormalities could lead to self-generated mental actions—such as inner speech—being misperceived as externally-generated, which could underpin the phenomenon of AVH.^1^^,^^2^^,^^21–24^

Given this theoretical context, the pattern of results exhibited by the SZAVH+ group in the present study is noteworthy. To reiterate, rather than the normative pattern of inner SIS of the auditory cortex,^25^^,^^26^^,^^48–54^ data from the hallucinating patients actually showed inner-speaking induced *enhancement*. Regarding the functional significance of this result: the amplitude of the N1 component is believed to be an index of auditory salience, as demonstrated by the fact that loud sounds elicit larger N1-amplitudes than do soft sounds.^55^^,^^56^ This suggests that while inner speech has the effect of dampening the salience of predicted auditory sounds in healthy controls, it actually has the effect of *enhancing* the salience of these sounds in people with schizophrenia who are currently experiencing AVH. This enhancement could, we suggest, lead to confusion as to whether sounds in the environment are internally or externally generated, which could lead to the misperception of internally-generated inner speech as externally-generated overt speech, and hence the experience of AVH.^57^

The present study replicates and extends the previous studies of Ford and Mathalon which used a similar “Talk-Listen” type task design^14^ to explore inner speech dysfunction in schizophrenia.^51^^,^^58^ In these studies, patients with schizophrenia and healthy controls were instructed to imagine a series of statements that resembled those commonly reported in AVH (eg, “That was really stupid”) while listening to auditory probes (eg, a speech sound /ba/). While healthy controls showed a smaller N1 in the inner speech condition relative to the passive condition, patients with schizophrenia exhibited no N1 differences between the 2 conditions, a result consistent with a deficit of “inner SIS.” While these studies were seminal in suggesting “inner SIS” deficits in schizophrenia, the present study provides stronger evidence that the deficits are a consequence of corollary discharge dysfunction, due to 2 methodological advantages, namely: (1) we tightly time-locked the inner-speech to the audible sound, and (2) we included a Mismatch condition for comparison, thus minimizing the possibility that the apparent deficits in “inner SIS” were due to attentional deficits in the clinical participants. Given this, the present study provides perhaps the strongest evidence to date that inner speech deficits are an etiological factor for AVH in patients with schizophrenia spectrum disorders.

While the SZAVH− patients did not show the same enhancement of N1-amplitude in the Match condition as did the SZAVH+ patients, they nonetheless did not exhibit the normative pattern typical of healthy controls. Specifically, while there was no difference in N1-amplitude between the Match and Passive conditions in this group, they did, however, show N1-suppression in the *Mismatch* condition. One possible interpretation for the lack of difference between the Match vs. Passive conditions is that the non-hallucinating patients were, in a sense, “intermediate” between the controls and hallucinating patients; that is, between the inner-speaking induced suppression exhibited by the controls and the inner speaking-induced enhancement exhibited by the hallucinating patients. This interpretation is consistent with the significant correlation that was observed between the severity of patients’ AVH (as assessed with the PSYRATS) and N1-suppression score when collapsing across the 2 clinical groups.

With regards to the observed difference between the Match and Mismatch conditions in the SZAVH− group: it is notable that the pattern of N1-amplitudes was the same in both patient groups; that is, N1-amplitude was larger in the Match condition compared to the Mismatch condition in both the SZAVH+ and SZAVH− groups, which was the opposite pattern to that exhibited by the HC participants. This result suggests that the evoked response to audible sounds is abnormally “boosted” in people with schizophrenia-spectrum disorders, but only in the case where the sounds match the content of their inner speech. While this “Match-Boost” process was observed regardless of current AVH status in our sample, our data suggest that the magnitude and/or impact of the “Match-Boost” response was greatest in SZAVH+ participants, where it was sufficient to cause the evoked response in the Match condition to exceed that of the Passive condition (in which no inner speech was produced). That is, in patients with current experience of AVH, the impact of the “Match-Boost” process was large enough to reverse the typical pattern of inner-speech-induced suppression. Thus we speculate that while the “Match-Boost” process may index susceptibility to schizophrenia-spectrum disorders in general, at more extreme levels it may index a tendency for AVH specifically. Testing this hypothesis with a longitudinal experimental design may be a worthwhile aim for future research.

As for the reason why N1-amplitude was reduced in the Mismatch condition relative to the Passive condition in the SZAVH− group: we believe that this result may reflect a between-condition difference in prediction error. Specifically, we suggest that there is a greater degree of prediction error in the Passive condition (in which there is no prediction) compared to the Mismatch condition (in which there is an imperfect prediction), and that this difference is reflected in the observed differences in N1-amplitude.^59^ Consistent with this idea, we note that the HC group also showed a (non-significant) trend in this direction in the present study, which also mirrors the trend we have observed in our previous studies, in which the N1 in the Mismatch condition was observed to be intermediate between the Match and Passive conditions.^27^ It is also interesting to note that the severity of patients’ PSYRATS Auditory Hallucinations score was negatively correlated with their “Mismatch minus Passive” difference score (see Figure 2(vi)), and that this correlation remained significant even when restricting this analysis to just the SZAVH+ participants. This result suggests that having a smaller (less negative) N1 response to the Mismatch condition (relative to the Passive condition) may be somehow associated with reduced susceptibility to AVH (in the SZAVH− group) or a reduction in the severity of auditory-verbal hallucinations (in the SZAVH+ group), possibly because a smaller N1-response reflects a reduction in the perceived salience of mispredicted (or imperfectly predicted) events, while AVH may reflect an increase in the perceived salience of expected events (ie, inner speech). However, when making these speculations, it should be noted that the 2 clinical groups did had somewhat different diagnostic profiles. Specifically, as shown in Table 1, the SZAVH+ group had a somewhat higher proportion of schizophrenia patients compared to the SZAVH− group, which had a more mixed profile, with an increased proportion of schizoaffective, brief psychotic and delusional disorder patients. The extent to which these differences in diagnostic profile could underpin the observed differences in inner SIS are unclear, and would be a worthwhile focus for future studies.

We have conceptualized the results of the present study in terms of a dysfunction in the efference copy/corollary discharge mechanisms associated with inner speech production. According to this view, this corollary-discharge dysfunction is conceptually similar, or perhaps even identical, to the dysfunction associated with the production of overt actions, with the phenomenon of SIS being a case in point. It is interesting to consider how these findings might relate to another influential account of hallucinations, namely the “strong priors” hypothesis, in which overweighted prior expectations come to dominate sensory input, ultimately leading to “percepts with no corresponding stimuli at all.”^60^ A recent paper by Leptourgos and Corlett^61^ made the interesting suggestion that these 2 accounts (ie, the “corollary-discharge” and “strong priors” hypotheses) may in fact be causally related. This theory, which has some recent empirical support,^62^ suggests that a failure of corollary-discharge-related mechanisms to suppress self-generated inputs may result in a “compensatory enhancement of allo-centric priors (p. 1)” that are involved in generating causal models of the world. In other words, corollary-discharge dysfunctions may ultimately *cause* the overweighting of priors that ultimately underpin the experience of hallucinations. While the current study does not provide a direct test of this theory, in our opinion it is a plausible idea, and one which is consistent with previous suggestions that corollary-discharge abnormalities might be expected to have significant downstream effects on the brains of people with psychosis (eg, relating to the dopamine system), given their centrality to nervous-system function.^63^

A question that naturally arises given the purely mental nature of the experimental task is whether the observed between-group differences might be due to the clinical participants being less engaged with the task than the healthy controls. We think that this possibility is unlikely, for if the clinical participants were not engaging in the task (ie, not producing the required inner syllable at the designated time), then every trial would essentially be a “Passive” trial, and we would not expect to see any differences in N1-amplitude between the Match, Mismatch, and Passive conditions. However, this was not the case for either of the 2 clinical groups in the current study; that is, between-condition differences in N1-amplitude were observed in all 3 participant groups, but the pattern of these between-condition differences differed markedly between the groups.

With regards to the P2 results: the main finding was while the HC participants exhibited increased P2-amplitude in the Match condition, relative to the Mismatch and Passive conditions, there were no between-condition differences in P2-ampliutde in either of the 2 SZ groups. We have previously reported P2-amplitude reductions in the Mismatch condition (relative to Match) in healthy control participants,^25^^,^^27^ and have argued that this P2-reduction may reflect a “cognitive prediction error” (ie, the conscious recognition of a difference in content between the inner and audible syllables in the Mismatch condition), as opposed to the lower-level “sensory” predictions indexed by the N1.^27^ If this speculation is correct, then the fact that the 2 SZ groups did not exhibit this P2 reduction in the Mismatch condition may indicate that they either did not recognize the mismatch between the inner or audible syllables, or—perhaps more likely—they did not attend as strongly to the stimuli, and did not make as strong “cognitive predictions” as did the HC participants.

With regards to the P3 results: the main finding was that while P3-amplitude was reduced in the Passive condition (relative to the Match condition) in the HC participants, P3-amplitude was reduced in the Match condition (relative to both the Passive and Mismatch conditions) in the SZAVH− participants, with no between-condition differences in P3-amplitude in the SZAVH+ participants. We have previously reported that the Passive condition elicits smaller P3-amplitues than both the Match and Mismatch conditions in HC participants.^25^ This may be due to diminished attentional resources being allocated to the stimuli in this condition, relative to the 2 inner speech conditions.^64^^,^^65^ Relatedly, we have previous argued that the reduction in P3 may reflect the HC participants making a mental response in the inner speech conditions (a “template-matching” response, perhaps, along the lines of whether the audible syllable matched the inner syllable) that they did not make in the Passive condition. If this is the case, then the fact that the 2 SZ groups did not exhibit this reduction in P3-amplitude in the Passive condition may suggest that they did not engage in this “template-matching response” (or at least not as strongly as the HC participants), a possibility which would be consistent with our speculative explanation regarding the P2-component. With regards to why the Match condition would show reduced P3-amplitude in the SZAVH− participants: while this result was not predicted, and we do not have a definitive explanation, we do note that the Match waveform was more negative than both Mismatch and Passive waveforms from around 170 ms post-stimulus until the end of the epoch (see Figure 4). In other words, the apparent “reduction” in P3-amplitude in the Match condition might have disappeared if we had baseline-corrected to the P2-window (as opposed to the pre-stimulus interval), and thus it is possible that the apparent P3-reduction in the Match condition ultimately reflects a reduction in P2.

### Limitations

The study did have some limitations. Firstly, with regards to the AVH status of the schizophrenia spectrum patients: while none of the SZAVH− participants were hallucinating at the time of testing (and had not hallucinated in the past week, as per the criteria of the PANSS^29^), many of these participants has a past history of AVH, as shown in Table 1. This is unsurprising, given that AVH are the most common psychotic symptom in schizophrenia spectrum disorders, with an estimated prevalence of around 75% in schizophrenia.^66^ It would be highly informative (though logistically challenging) to repeat this experimental protocol on a sample of patients without any history of AVH. Secondly, there are several different “classes” or “subtypes” of AVH that commonly occur in schizophrenia spectrum disorders, including audible thoughts, voices commenting, command hallucinations, and others.^66–70^ Due to the limited sample size of the SZAVH+ group, we were unable to differentiate between these different classes of AVH. Thus, while the results of this study provide support for the hypothesis that deficits in inner SIS may be associated with AVH *in general*, it is not clear whether this applies to all classes of AVH equally. It is possible (perhaps likely) that certain classes of AVH—such as audible thoughts, which most clearly seem to reflect the misperception of inner speech as external speech^71^—are particularly strongly associated with deficits in “inner SIS.” Finally, it should be reiterated that in order to recruit the required sample size, it was necessary to collect the data at 2 different testing sites (in Australia and Hong Kong), which had different EEG systems. We do not believe that this had a significant influence on the results, for 2 reasons: (1) we controlled for testing site by including it as a nuisance covariate in the analyses; and (2) the key dependent variable (N1-suppression) was within-subjects in nature. Nevertheless, it would be worthwhile replicating these results in a participant sample tested on a single EEG system.

In conclusion, the present study provides perhaps the strongest evidence to date that AVH commonly associated with schizophrenia spectrum disorders are related to an abnormality in the normatively suppressive mechanisms associated with the production of inner speech: a phenomenon we have dubbed inner SIS. Our hope is that the phenomenon of “inner SIS” may have utility as a neurophysiological biomarker for schizophrenia-spectrum disorders generally and/or AVH specifically, which could complement existing neurophysiological biomarkers,^72^^,^^73^ and potentially represent a useful target for treatment response and clinical outcome. It may also have utility in predicting transition to psychosis in at-risk individuals,^74^ which is one of the key aims of the Accelerating Medicines Partnership—Schizophrenia program that is currently ongoing in collaboration with the National Institutes of Health.^76^

## Supplementary Material

ImaginatronSZ_SupplementaryMaterials_R2_sbaf167
