# Supplementary material for: Corollary Discharge Dysfunction to Inner Speech and its Relationship to Auditory Verbal Hallucinations in Patients with Schizophrenia Spectrum Disorders
Source: Schizophr Bull. 2025 Oct 21;52(5):sbaf167. doi: 10.1093/schbul/sbaf167 (PMC13645647; doi:10.1093/schbul/sbaf167)
Supplement: Supplementary file 1 [file imaginatronsz_supplementarymaterials_r2_sbaf167.docx]

***Supplementary Statistical Analyses – Linear Mixed Models***

We conducted supplementary statistical analyses in R version 4.5.0. Linear mixed models were used to examine associations between variables, following the procedure outlined in (Duggirala et al., 2024). Mean N1 amplitude represented the outcome variable, *Group* (3 levels; HC vs SZAVH+ vs SZAVH-, dummy-coded) was a between-group fixed effect, and *Condition* (3 levels; Passive vs Match vs Mismatch, dummy-coded) was a within-subjects fixed effect. Given data was conducted at two sites, we controlled for *Location* (2 levels, Australia vs Hong Kong). A random intercept was included to capture variability in participants’ average N1 amplitude. We investigated whether N1 amplitude varied according to *Group* and *Condition* (over and above *Location*), as described in the above model. Next, we investigated whether the effects of interest were maintained with the inclusion of potential covariates (*Age, Sex*), and whether the associations varied across levels of these covariates (*Age, Sex, Location*).

We then sought to investigate whether the association between *Condition* and *Group* in the clinical groups varied according to clinical variables of interest. Given data on clinical variables (PANSS Positive Total, PANSS Negative Total, PANSS General Total, PSYRATS Delusions score) was only relevant to the SZAVH+ and SZAVH- groups (not the healthy controls), we limited this analysis to the two clinical groups. As these variables are conceptually and statistically interrelated, we elected to examine the impact of each variable on the model individually. For these analyses, we modelled the effect of *Group* (SZAVH+ and SZAVH-) and condition on N1 amplitude as above (controlling for *Location*). We first investigated whether adding the covariate to the model improved model fit. A separate baseline model was estimated for each clinical variable to ensure that the models were testing equivalent data (i.e., taking into account missing data on the clinical variables). We then investigated (1) whether the effects of interest were maintained with the inclusion of clinical variables, and (2) whether the associations varied across levels of these variables. All continuous clinical variables were mean-centered.

For the PSYRATS Auditory Hallucinations score, all participants in the SZAVH- group scored zero, while those in the SZAVH+ group varied in terms of their score. For this variable, we elected to investigate the association between severity of auditory-verbal hallucinations and (1) N1-suppression: calculated as the difference in N1-amplitude between the Match and Passive conditions, and (2) the difference in N1-amplitude between the Mismatch and Passive conditions, using a Spearman’s Rank Order Correlation (see Results section, main manuscript).

Finally, we tested the association between the other clinical variables (PANSS Positive Total, PANSS Negative Total, PANSS General Total, PSYRATS Delusions score), and N1-suppression. For these analyses, we excluded healthy controls (who were not administered the clinical scales), and conceptualized the SZAVH+ and SZAVH- participants as constituting a single group, differentiated on severity of clinical variables. For the aforementioned variables, we first conducted a multiple regression analysis, investigating whether a model with all of these variables included simultaneously predicted significant variance in the N1 amplitude difference score between the Match and Passive conditions.

**Results.**

We first investigated the impact of *Condition* and *Group* on N1 amplitude with Healthy Controls (*Group*) and Passive (*Condition*) as the reference groups, controlling for *Location* (Supplementary Table A). A Type III ANOVA using Satterthwaite’s approximation revealed a significant main effect for *Location* F(1,138) = 8.87, *p*=.003 and a significant *Condition x Group* interaction F(4,278) = 5.27, *p*<.001. Investigation of fixed effects indicated that the significant *Condition x Group* interaction was driven by a Match x SZAVH+ interaction. Simple slopes analyses revealed that AVH+ participants showed a larger (more negative) response in the Match condition (mean = -4.52, SE = 0.54) than in the Passive condition (mean = -3.22, SE = 0.54; t(278) = 3.69, *p=*.0008).

| **Supplementary Table A.** Linear Mixed Effects Model for N1, reference group = Healthy Controls, reference condition = Passive | | | | |  |
| --- | --- | --- | --- | --- | --- |
|  | Est | SE | df | t value | p-value |
| Fixed effects |  |  |  |  |  |
| Intercept | -5.92 | 0.74 | 167.91 | -7.97 | <.001 |
| Condition |  |  |  |  |  |
| Passive (reference) |  |  |  |  |  |
| Match | 0.64 | 0.40 | 278.00 | 1.61 | 0.108 |
| Mismatch | 0.12 | 0.40 | 278.00 | 0.30 | 0.765 |
| Group |  |  |  |  |  |
| Healthy Controls (reference) |  |  |  |  |  |
| SZAVH+ | 1.75 | 0.81 | 186.02 | 2.16 | 0.032 |
| SZAVH- | -0.26 | 0.85 | 186.21 | -0.3 | 0.759 |
| Location |  |  |  |  |  |
| Australia |  |  |  |  |  |
| Hong Kong | 1.91 | 0.64 | 138.00 | 2.98 | 0.003 |
| Condition x Group |  |  |  |  |  |
| Passive x Healthy Controls (reference) |  |  |  |  |  |
| Match x SZAVH+ | -1.94 | 0.53 | 278.00 | -3.65 | 0.003 |
| Mismatch x SZAVH+ | -0.64 | 0.53 | 278.00 | -1.20 | 0.232 |
| Match x SZAVH- | -0.91 | 0.56 | 278.00 | -1.62 | 0.107 |
| Mismatch x SZAVH- | 0.83 | 0.56 | 278.00 | 1.48 | 0.141 |
|  |  |  |  |  |  |
| Random effects: |  |  |  |  |  |
| Groups | Name | Variance | SD |  |  |
| Participants | Intercept | 12.26 | 3.5 |  |  |
| Residual |  | 3.41 | 1.84 |  |  |
| Number of observations: 426, participants: 142 | | | |  |  |

With healthy controls (*Group*) and Mismatch (*Condition*) as the reference, the model investigating the impact of *Condition* and *Group* on N1 amplitude (controlling for *Location*) revealed significant *Condition x Group* interactions for Passive x SZAVH- and Match x SZAVH- (See Supplementary Table B). Simple slopes analyses revealed that SZAVH- participants showed a smaller (less negative) response in the Mismatch condition (mean = -4.28, SE = 0.61) than in the Passive condition (mean = -5.22, SE = 0.61; t(278) = 2.40, *p=*.045), and a larger response in the Match (mean = -5.49, SE = 0.61) condition than in the Mismatch condition (mean = -4.28, SE = 0.61; t(278) = 3.07, *p*=.007).

| **Supplementary Table B.** Linear Mixed Effects Model for N1, reference group = Healthy Controls, reference condition = Mismatch | | | | |  |
| --- | --- | --- | --- | --- | --- |
|  | Est | SE | df | t value | p-value |
| Fixed effects |  |  |  |  |  |
| Intercept | -5.8 | 0.74 | 167.91 | -7.81 | <.001 |
| Condition |  |  |  |  |  |
| Mismatch (Reference) |  |  |  |  |  |
| Passive | -0.11 | 0.4 | 278 | -0.3 | 0.765 |
| Match | 0.52 | 0.4 | 278 | 1.31 | 0.190 |
| Group |  |  |  |  |  |
| Healthy Controls (reference) |  |  |  |  |  |
| SZAVH+ | 1.11 | 0.81 | 186.02 | 1.38 | 0.171 |
| SZAVH- | 0.57 | 0.85 | 186.22 | 0.67 | 0.506 |
| Location |  |  |  |  |  |
| Australia |  |  |  |  |  |
| Hong Kong | 1.91 | 0.64 | 138 | 2.98 | 0.003 |
| Condition x Group |  |  |  |  |  |
| Passive x Healthy Controls (reference) |  |  |  |  |  |
| Passive x SZAVH+ | 0.64 | 0.53 | 278 | 1.20 | 0.232 |
| Match x SZAVH+ | -1.3 | 0.53 | 278 | -2.45 | 0.015 |
| Passive x SZAVH- | -0.83 | 0.56 | 278 | -1.48 | 0.141 |
| Match x SZAVH- | -1.73 | 0.56 | 278 | -3.09 | 0.002 |
|  |  |  |  |  |  |
| Random effects: |  |  |  |  |  |
| Groups | Name | Variance | SD |  |  |
| Participants | Intercept | 12.26 | 3.5 |  |  |
| Residual |  | 3.41 | 1.84 |  |  |
| Number of observations: 426, participants: 142 | | | |  |  |

Next, we tested whether the *Condition x Group* interaction was maintained with the addition of covariates. In addition to location, we tested models that included *Age* and *Sex*. A Type III ANOVA using Satterthwaite’s approximation revealed a significant main effect for *Age* such that participants who were older showed a smaller (less negative) response F(1, 137) = 10.17, *p*=.002 (Supplementary Table C). The *Condition x Group* interaction maintained significance with the inclusion of *Age*, F(4, 278) = 5.27, *p*=0.004.

| **Supplementary Table C.** Linear Mixed Effects Model for N1 Including Age, reference group = Healthy Controls, reference condition = Passive | | | | |  |
| --- | --- | --- | --- | --- | --- |
|  | Est | SE | df | t value | p-value |
| Fixed effects |  |  |  |  |  |
| Intercept | -5.90 | 0.72 | 168.69 | -8.18 | <.001 |
| Condition |  |  |  |  |  |
| Passive (Reference) |  |  |  |  |  |
| Match | 0.64 | 0.4 | 278 | 1.61 | 0.108 |
| Mismatch | 0.12 | 0.4 | 278 | 0.30 | 0.765 |
| Group |  |  |  |  |  |
| Healthy Controls (reference) |  |  |  |  |  |
| SZAVH+ | 1.67 | 0.79 | 187.84 | 2.12 | 0.035 |
| SZAVH- | -0.54 | 0.83 | 187.42 | -0.65 | 0.515 |
| Age | -0.08 | 0.24 | 137 | -3.19 | 0.002 |
| Location |  |  |  |  |  |
| Australia |  |  |  |  |  |
| Hong Kong | 2.07 | 0.62 | 137 | 3.32 | 0.001 |
| Condition x Group |  |  |  |  |  |
| Passive x Health Controls (reference) |  |  |  |  |  |
| Match x SZAVH+ | -1.94 | 0.53 | 278 | -3.65 | <.001 |
| Mismatch x SZAVH+ | -0.64 | 0.53 | 278 | -1.20 | 0.232 |
| Match x SZAVH- | -0.91 | 0.56 | 278 | -1.62 | 0.107 |
| Mismatch x SZAVH- | 0.83 | 0.56 | 278 | 1.48 | 0.141 |
|  |  |  |  |  |  |
| Random effects: |  |  |  |  |  |
| Groups | Name | Variance | SD |  |  |
| Participants | Intercept | 11.42 | 3.4 |  |  |
| Residual |  | 3.41 | 1.85 |  |  |
| Number of observations: 426, participants: 142 | | | |  |  |

A Type III ANOVA using Satterthwaite’s approximation revealed a significant main effect for *Sex*, such that female participants showed a larger (more negative) N1 response overall F(1,137) = 4.00, *p*=.047 (Supplementary Table D). The *Condition x Group* interaction maintained significance with the inclusion of *Sex*, F(4, 278) = 5.27, *p*=0.004.

| **Supplementary Table D.** Linear Mixed Effects Model for N1 Including Sex, reference group = Healthy Controls, reference condition = Passive | | | | |  |
| --- | --- | --- | --- | --- | --- |
|  | Est | SE | df | t value | p-value |
| Fixed effects |  |  |  |  |  |
| Intercept | -6.57 | 0.80 | 161.85 | -8.17 | <.001 |
| Condition |  |  |  |  |  |
| Passive (Reference) |  |  |  |  |  |
| Match | 0.64 | 0.4 | 278 | 1.61 | 0.108 |
| Mismatch | 0.12 | 0.4 | 278 | 0.30 | 0.765 |
| Group |  |  |  |  |  |
| Healthy Controls (reference) |  |  |  |  |  |
| SZAVH+ | 1.75 | 0.80 | 185.74 | 2.19 | 0.030 |
| SZAVH- | -0.32 | 0.84 | 185.84 | -0.39 | 0.696 |
| Sex |  |  |  |  |  |
| Male (reference) |  |  |  |  |  |
| Female | 1.22 | 0.61 | 137 | -2.00 | 0.047 |
| Location |  |  |  |  |  |
| Australia |  |  |  |  |  |
| Hong Kong | 1.95 | 0.64 | 137 | 3.08 | 0.003 |
| Condition x Group |  |  |  |  |  |
| Passive x Healthy Controls (reference) |  |  |  |  |  |
| Match x SZAVH+ | -1.94 | 0.53 | 278 | -3.65 | <.001 |
| Mismatch x SZAVH+ | -0.64 | 0.53 | 278 | -1.20 | 0.232 |
| Match x SZAVH- | -0.91 | 0.56 | 278 | -1.62 | 0.107 |
| Mismatch x SZAVH- | 0.83 | 0.56 | 278 | 1.48 | 0.141 |
|  |  |  |  |  |  |
| Random effects: |  |  |  |  |  |
| Groups | Name | Variance | SD |  |  |
| Participants | Intercept | 11.97 | 3.46 |  |  |
| Residual |  | 3.41 | 1.85 |  |  |
| Number of observations: 426, participants: 142 | | | |  |  |

A Type III ANOVA using Satterthwaite’s approximation revealed a significant main effect for *Location* such that participants in Hong Kong showed a smaller (less negative) response overall F(1,136) = 11.43, *p*=.001 (Supplementary Table A). As noted above, the *Condition* x *Group* interaction remained significant with the inclusion of location in the model.

Next, we tested whether the *Condition* x *Group* interaction varied over levels of covariates. There were no significant three-way interactions with group and condition of *Age* F(4,272) = 0.24, *p* = 0.915, *Sex* F(4, 272) = 0.64, *p*=0.752 or *Location* F(4,272) = 1.37, *p* = 0.245 (see Supplementary Tables E, F and G).

**Supplementary Table E.** Linear Mixed Effects Model for N1 Including Interaction with Age, reference group = Healthy Controls, reference condition = Passive

|  | Est | SE | df | t value | p-value |
| --- | --- | --- | --- | --- | --- |
| Fixed effects |  |  |  |  |  |
| Intercept | -5.85 | 0.73 | 166.11 | -7.98 | <.001 |
| Condition |  |  |  |  |  |
| Passive (Reference) |  |  |  |  |  |
| Match | 0.68 | 0.40 | 272 | 1.68 | 0.094 |
| Mismatch | 0.11 | 0.40 | 272 | 0.28 | 0.781 |
| Group |  |  |  |  |  |
| Healthy Controls (reference) |  |  |  |  |  |
| SZAVH+ | 1.62 | 0.79 | 185.53 | 2.05 | 0.042 |
| SZAVH- | -0.51 | 0.84 | 185.75 | -0.61 | 0.544 |
| Sex | -0.11 | 0.05 | 185.78 | -2.33 | 0.021 |
| Location |  |  |  |  |  |
| Australia |  |  |  |  |  |
| Hong Kong | 2.06 | 0.64 | 135 | 3.24 | 0.002 |
| Condition x Group |  |  |  |  |  |
| Passive x Healthy Controls (reference) |  |  |  |  |  |
| Match x SZAVH+ | -1.98 | 0.54 | 272 | -3.69 | <.001 |
| Mismatch x SZAVH+ | -0.64 | 0.54 | 272 | -1.19 | 0.237 |
| Match x SZAVH- | -0.97 | 0.57 | 272 | -1.7 | 0.090 |
| Mismatch x SZAVH- | 0.83 | 0.57 | 272 | 1.46 | 0.146 |
| Age x Condition |  |  |  |  |  |
| Age x Passive (reference) |  |  |  |  |  |
| Age x Match | -0.02 | 0.03 | 272 | -0.77 | 0.445 |
| Age x Mismatch | 0.01 | 0.03 | 272 | 0.14 | 0.886 |
| Age x Group |  |  |  |  |  |
| Age x Healthy Controls (reference) |  |  |  |  |  |
| Age x SVAH+ | 0.03 | 0.06 | 184.82 | 0.41 | 0.685 |
| Age x SVAH- | 0.06 | 0.07 | 185.6 | 0.89 | 0.374 |
| Age x Condition x Group |  |  |  |  |  |
| Age x Passive x Healthy Controls (reference) |  |  |  |  |  |
| Age x Match x SZAVH+ | 0.04 | 0.04 | 272 | 0.91 | 0.364 |
| Age x Mismatch x SZAVH+ | 0.02 | 0.04 | 272 | 0.44 | 0.660 |
| Age x Match x SZAVH- | 0.01 | 0.05 | 272 | 0.18 | 0.855 |
| Age x Mismatch x SZAVH- | 0.01 | 0.05 | 272 | -0.10 | 0.922 |
|  |  |  |  |  |  |
| Random effects: |  |  |  |  |  |
| Groups | Name | Variance | SD |  |  |
| Participants | Intercept | 11.49 | 3.39 |  |  |
| Residual |  | 3.47 | 1.86 |  |  |
| Number of observations: 426, participants: 142 | |  |  |  |  |

**Supplementary Table F.** Linear Mixed Effects Model for N1, including Interactions with Gender, reference group = Healthy Controls, reference condition = Passive

|  | Est | SE | df | t value | p-value |
| --- | --- | --- | --- | --- | --- |
| Fixed effects |  |  |  |  |  |
| Intercept | -6.4 | 0.97 | 171.79 | -6.61 | <.001 |
| Condition |  |  |  |  |  |
| Passive (Reference) |  |  |  |  |  |
| Match | 0.57 | 0.57 | 272 | 1.00 | 0.320 |
| Mismatch | 0.27 | 0.57 | 272 | 0.47 | 0.641 |
| Group |  |  |  |  |  |
| Healthy Controls (reference) |  |  |  |  |  |
| SZAVH+ | 2.02 | 1.14 | 183.79 | 1.77 | 0.079 |
| SZAVH- | -1.31 | 1.24 | 184.24 | -1.06 | 0.292 |
| Sex |  |  |  |  |  |
| Female (reference) |  |  |  |  |  |
| Male | 0.76 | 1.20 | 184.15 | 0.64 | 0.523 |
| Location |  |  |  |  |  |
| Australia (reference) |  |  |  |  |  |
| Hong Kong | 2.04 | 0.64 | 135 | 3.21 | 0.002 |
| Condition x Group |  |  |  |  |  |
| Passive x Healthy Controls (reference) |  |  |  |  |  |
| Match x SZAVH+ | -2.10 | 0.77 | 272 | -2.74 | 0.007 |
| Mismatch x SZAVH+ | -0.76 | 0.77 | 272 | -1.00 | 0.319 |
| Match x SZAVH- | -0.83 | 0.83 | 272 | -0.99 | 0.322 |
| Mismatch x SZAVH- | 0.24 | 0.83 | 272 | 0.28 | 0.777 |
| Gender x Condition |  |  |  |  |  |
| Female x Passive (reference) |  |  |  |  |  |
| Male x Match | 0.14 | 0.80 | 272 | 0.17 | 0.863 |
| Male x Mismatch | -0.29 | 0.80 | 272 | -0.36 | 0.718 |
| Gender x Group |  |  |  |  |  |
| Female x Healthy Controls (reference) |  |  |  |  |  |
| Male x SVAH+ | -0.51 | 1.60 | 184 | -0.32 | 0.749 |
| Male x SVAH- | 1.78 | 1.69 | 184.22 | 1.05 | 0.294 |
| Gender x Condition x Group |  |  |  |  |  |
| Female x Passive x Healthy Controls (reference) |  |  |  |  |  |
| Male x Match x SZAVH+ | 0.30 | 1.07 | 272 | 0.28 | 0.778 |
| Male x Mismatch x SZAVH+ | 0.25 | 1.07 | 272 | 0.23 | 0.815 |
| Male x Match x SZAVH- | -0.15 | 1.13 | 272 | -0.14 | 0.893 |
| Male x Mismatch x SZAVH- | 1.07 | 1.13 | 272 | 0.94 | 0.348 |
|  |  |  |  |  |  |
| Random effects: |  |  |  |  |  |
| Groups | Name | Variance | SD |  |  |
| Participants | Intercept | 11.86 | 3.44 |  |  |
| Residual |  | 3.46 | 1.86 |  |  |
| Number of observations: 426, participants: 142 | |  |  |  |  |

**Supplementary Table G.** Linear Mixed Effects Model for N1, Including interactions with Location reference group = Healthy Controls, reference condition = Passive

| Term | Est | | SE | df | t value | p-value |
| --- | --- | --- | --- | --- | --- | --- |
| Fixed effects | |  |  |  |  |  |
| Intercept | | -6.61 | 1.06 | 182.17 | -6.26 | <.001 |
| Condition | |  |  |  |  |  |
| Passive (Reference) | |  |  |  |  |  |
| Match | | 0.92 | 0.69 | 272 | 1.34 | 0.181 |
| Mismatch | | 0.34 | 0.69 | 272 | 0.5 | 0.619 |
| Group | |  |  |  |  |  |
| Healthy Controls (reference) | |  |  |  |  |  |
| SZAVH+ | | 3.14 | 1.35 | 182.17 | 2.32 | 0.021 |
| SZAVH- | | -0.62 | 1.47 | 182.17 | -0.42 | 0.671 |
| Location | |  |  |  |  |  |
| Australia | |  |  |  |  |  |
| Hong Kong | | 2.93 | 1.28 | 182.17 | 2.28 | 0.024 |
| Condition x Group | |  |  |  |  |  |
| Passive x Healthy Controls (reference) | |  |  |  |  |  |
| Match x SZAVH+ | | -2.71 | 0.88 | 272 | -3.07 | 0.002 |
| Mismatch x SZAVH+ | | -0.42 | 0.88 | 272 | -0.47 | 0.638 |
| Match x SZAVH- | | -1.06 | 0.96 | 272 | -1.11 | 0.268 |
| Mismatch x SZAVH- | | 2.08 | 0.96 | 272 | 2.18 | 0.030 |
| Location x Condition | |  |  |  |  |  |
| Australia x Passive (reference) | |  |  |  |  |  |
| Hong Kong x Match | | -0.42 | 0.84 | 272 | -0.50 | 0.620 |
| Hong Kong x Mismatch | | -0.33 | 0.84 | 272 | -0.40 | 0.693 |
| Location x Group | |  |  |  |  |  |
| Australia x Healthy Controls (reference) | |  |  |  |  |  |
| Hong Kong x SZAVH+ | | -2.19 | 1.68 | 182.17 | -1.30 | 0.195 |
| Hong Kong x SZAVH- | | 0.57 | 1.80 | 182.17 | 0.32 | 0.749 |
| Location x Condition x Group | |  |  |  |  |  |
| Australia x Passive x Healthy Controls (reference) | |  |  |  |  |  |
| Hong Kong x Match x SZAVH+ | | 1.22 | 1.10 | 272 | 1.11 | 0.266 |
| Hong Kong x Mismatch x SZAVH+ | | -0.41 | 1.10 | 272 | -0.37 | 0.709 |
| Hong Kong x Match x SZAVH- | | 0.23 | 1.17 | 272 | 0.19 | 0.847 |
| Hong Kong x Mismatch x SZAVH- | | -1.91 | 1.17 | 272 | -1.63 | 0.104 |
|  | |  |  |  |  |  |
| Random effects: | |  |  |  |  |  |
| Groups | | Name | Variance | SD |  |  |
| Participants | | Intercept | 12.27 | 3.50 |  |  |
| Residual | |  | 3.32 | 1.82 |  |  |
| Number of observations: 426, participants: 142 | | |  |  |  |  |

*Association with clinical variables.*

We first investigated the impact of *Condition* and *Group* on N1 amplitude in the clinical groups only, with SZAVH- (*Group*) and Passive (*Condition*) as the reference groups, controlling for *Location* (See Supplementary Table H). A Type III ANOVA using Satterthwaite’s approximation revealed a significant main effect for *Location*, F(1,99) = 4.19, *p*=.043, a significant main effect for *Condition*, F(2,198) = 6.48, *p*=0.002, and a significant *Condition x Group* interaction, F(2,198) = 3.35, *p*=.037. Investigation of fixed effects indicated that a significant Mismatch x SZAVH+ interaction (Est = -1.46, SE = 0.58, p=0.013) and a marginally significant Match x SZAVH+ interaction (Est = -1.04, SE = 0.58, p=0.076). Simple slopes analyses revealed that SZAVH+ participants showed a larger (more negative) response in the Match condition (mean = -4.48, SE = 0.57) than in the Passive condition (mean = -3.18, SE = 0.57; t(202) = -3.14, *p=*.001). Further, SZAVH+ participants showed a larger (more negative) response in the Match condition (mean = -4.48, SE = 0.57) than Mismatch condition (mean = -3.70, SE = 0.57; t(202) = 0.78, *p*=0.048). SZAVH- participants showed a larger (more negative) response in the Passive condition (mean = -5.17, SE = 0.64) than Mismatch condition (mean = -4.23, SE – 0.64; t(202) = 2.16, *p*=0.032). SZAVH- participants showed a larger (more negative) response in the Match condition (mean = -5.44, SE = 0.64) than the Mismatch condition (mean = --4.23, SE = 0.64; *t*(202)2.76, *p*=0.006)

| **Supplementary Table H.** Linear Mixed Effects Model for N1 in clinical groups, reference group = SZAVH-, reference condition = passive | | | | |  |
| --- | --- | --- | --- | --- | --- |
|  | Est | SE | df | t value | p-value |
| Fixed effects |  |  |  |  |  |
| Intercept | -5.97 | 0.80 | 120.74 | -7.43 | <.001 |
| Condition |  |  |  |  |  |
| Passive (reference) |  |  |  |  |  |
| Match | -0.26 | 0.43 | 198.00 | -0.61 | 0.545 |
| Mismatch | 0.95 | 0.43 | 198.00 | 2.18 | 0.030 |
| Group |  |  |  |  |  |
| SZAVH- (reference) |  |  |  |  |  |
| SZAVH+ | 1.99 | 0.83 | 138.92 | 2.34 | 0.018 |
| Location |  |  |  |  |  |
| Australia |  |  |  |  |  |
| Hong Kong | 1.59 | 0.78 | 99.00 | 2.05 | 0.043 |
| Condition x Group |  |  |  |  |  |
| Passive x SZAVH- (reference) |  |  |  |  |  |
| Match x SZAVH+ | -1.04 | 0.58 | 198.00 | -1.78 | 0.077 |
| Mismatch x SZAVH+ | -1.46 | 0.58 | 198.00 | -2.52 | 0.013 |
|  |  |  |  |  |  |
| Random effects: |  |  |  |  |  |
| Groups | Name | Variance | SD |  |  |
| Participants | Intercept | 12.64 | 3.56 |  |  |
| Residual |  | 4.14 | 2.03 |  |  |
| Number of observations: 297, participants: 99 | | | |  |  |

Next, we investigated the impact of including each of the clinical variables on the model.

*PANSS Positive score.* The inclusion of this variable in the model did not improve model fit (Baseline model: AIC = 1512, BIC = 1545, LL(288) = -747.0; model with covariate: AIC = 1514, BIC = 1551, LL = -747, Chi square difference (1) = 0.01, *p*-= 0.957, and the *Group* x *Condition* interaction retained significance: F(2,198) = 3.35, *p*=0.037. Consistent with this, PANSS Positive score was not a significant predictor of N1 amplitude: F(1, 99) = 0.01, p=0.957; Est = 0.01, SE = 0.07, t(99) = 0.05, *p*=0.957 (see Supplementary Table I). Further, the interaction between *PANSS Positive x Condition x Group* was not statistically significant: F(2, 198) = 0.06, *p*=0.940 (see Supplementary Table J).

| **Supplementary Table I.** Linear Mixed Effects Model for N1 in clinical groups, including PANSS Positive Score, reference group = SZAVH-, reference condition = Passive | | | | |  |
| --- | --- | --- | --- | --- | --- |
|  | Est | SE | df | t value | p-value |
| Fixed effects |  |  |  |  |  |
| Intercept | -5.97 | 0.82 | 119.95 | -7.30 | <.001 |
| Condition |  |  |  |  |  |
| Passive (reference) |  |  |  |  |  |
| Match | -0.26 | 0.43 | 198.00 | -0.61 | 0.545 |
| Mismatch | 0.95 | 0.43 | 198.00 | 2.18 | 0.030 |
| Group |  |  |  |  |  |
| SZAVH- (reference) |  |  |  |  |  |
| SZAVH+ | 1.97 | 0.93 | 129.70 | 2.12 | 0.036 |
| Location |  |  |  |  |  |
| Australia |  |  |  |  |  |
| Hong Kong | 1.60 | 0.79 | 99.00 | 2.03 | 0.045 |
| PANSS Positive Score | 0.01 | 0.07 | 99.00 | 0.05 | 0.957 |
| Condition x Group |  |  |  |  |  |
| Passive x SZAVH- (reference) |  |  |  |  |  |
| Match x SZAVH+ | -1.04 | 0.58 | 198.00 | -1.78 | 0.077 |
| Mismatch x SZAVH+ | -1.46 | 0.58 | 198.00 | -2.52 | 0.013 |
|  |  |  |  |  |  |
| Random effects: |  |  |  |  |  |
| Groups | Name | Variance | SD |  |  |
| Participants | Intercept | 12.64 | 3.56 |  |  |
| Residual |  | 4.14 | 2.03 |  |  |
| Number of observations: 297, participants: 99 | | | |  |  |

| **Supplementary Table J.** Linear Mixed Effects Model for N1 in clinical groups, including PANSS Positive Score interactions, reference group = SZAVH-, reference condition = Passive | | | | |  |
| --- | --- | --- | --- | --- | --- |
|  | Est | SE | df | t value | p-value |
| Fixed effects |  |  |  |  |  |
| Intercept | -5.99 | 0.87 | 125.85 | -6.89 | <.001 |
| Condition |  |  |  |  |  |
| Passive (reference) |  |  |  |  |  |
| Match | -0.19 | 0.52 | 198.00 | -0.37 | 0.709 |
| Mismatch | 0.93 | 0.52 | 198.00 | 1.81 | 0.072 |
| Group |  |  |  |  |  |
| SZAVH- (reference) |  |  |  |  |  |
| SZAVH+ | 2.06 | 0.96 | 138.83 | 2.153 | 0.033 |
| Location |  |  |  |  |  |
| Australia |  |  |  |  |  |
| Hong Kong | 1.60 | 0.79 | 99.00 | 2.02 | 0.046 |
| PANSS Positive Score | -0.01 | 0.13 | 137.92 | -0.04 | 0.969 |
| Condition x Group |  |  |  |  |  |
| Passive x SZAVH- (reference) |  |  |  |  |  |
| Match x SZAVH+ | -1.26 | 0.67 | 198.00 | -1.89 | 0.061 |
| Mismatch x SZAVH+ | -1.50 | 0.67 | 198.00 | -2.24 | 0.026 |
| PANSS Positive x Group |  |  |  |  |  |
| PANSS Positive x SZAVH - (reference) |  |  |  |  |  |
| PANSS Positive x SZAVH + | -0.02 | 0.16 | 138.71 | -0.10 | 0.922 |
| PANSS Positive x Condition |  |  |  |  |  |
| PANSS Positive x Passive (reference) |  |  |  |  |  |
| PANSS Positive x Match | 0.02 | 0.09 | 198.00 | 0.25 | 0.803 |
| PANSS Positive x Mismatch | -0.01 | 0.09 | 198.00 | -0.05 | 0.964 |
| PANSS Positive x Group x Condition |  |  |  |  |  |
| PANSS Positive x SZAVH - x Passive (reference) |  |  |  |  |  |
| PANSS Positive x SZAVH + x Match | 0.04 | 0.11 | 198.00 | 0.351 | 0.726 |
| PANSS Positive x SZAVH + Mismatch | 0.02 | 0.11 | 198.00 | 0.198 | 0.843 |
|  |  |  |  |  |  |
| Random effects: |  |  |  |  |  |
| Groups | Name | Variance | SD |  |  |
| Participants | Intercept | 12.65 | 3.56 |  |  |
| Residual |  | 4.12 | 2.03 |  |  |
| Number of observations: 297, participants: 99 | | | |  |  |

*PANSS Negative Score.* The inclusion of this variable in the model did not improve model fit (Baseline model: AIC = 1512, BIC = 1545, LL(288) = -747.0; Model with covariate: AIC = 1514, BIC = 1551, LL = -746.90, Chi square difference (1) = 0.21, *p*-= 0.644, and the *Group x Condition* interaction retained significance: F(2,198) = 3.34, *p*=0.037. Consistent with this, PANSS negative score was not a significant predictor of N1 amplitude F(1,99) = 0.21, p=0.644, Est = -0.02, SE = 0.06, t(99) = -0.46, *p*=0.645, See Supplementary Table K. Further, when included in the model, the interaction between *PANSS Negative x Condition x Group* was not statistically significant: F(2, 198) = 1.96, *p*=0.143, see Supplementary Table L.

| **Supplementary Table K.** Linear Mixed Effects Model for N1 in clinical groups, including PANSS Negative Score, reference group = SZAVH-, reference condition = Passive | | | | |  |
| --- | --- | --- | --- | --- | --- |
|  | Est | SE | df | t value | p-value |
| Fixed effects |  |  |  |  |  |
| Intercept | -5.80 | 0.89 | 116.47 | -6.54 | <.001 |
| Condition |  |  |  |  |  |
| Passive (reference) |  |  |  |  |  |
| Match | -0.26 | 0.43 | 198.00 | -0.61 | 0.545 |
| Mismatch | 0.95 | 0.43 | 198.00 | 2.18 | 0.030 |
| Group |  |  |  |  |  |
| SZAVH- (reference) |  |  |  |  |  |
| SZAVH+ | 1.87 | 0.87 | 134.72 | 2.15 | 0.033 |
| Location |  |  |  |  |  |
| Australia |  |  |  |  |  |
| Hong Kong | 1.42 | 0.86 | 99.00 | 1.65 | 0.101 |
| PANSS Negative Score | -0.03 | 0.06 | 99.00 | -0.46 | 0.645 |
| Condition x Group |  |  |  |  |  |
| Passive x SZAVH- (reference) |  |  |  |  |  |
| Match x SZAVH+ | -1.04 | 0.58 | 198.00 | -1.78 | 0.077 |
| Mismatch x SZAVH+ | -1.46 | 0.58 | 198.00 | -2.52 | 0.013 |
|  |  |  |  |  |  |
| Random effects: |  |  |  |  |  |
| Groups | Name | Variance | SD |  |  |
| Participants | Intercept | 12.61 | 3.56 |  |  |
| Residual |  | 4.14 | 2.03 |  |  |
| Number of observations: 297, participants: 99 | | | |  |  |

| **Supplementary Table L.** Linear Mixed Effects Model for N1 in clinical groups, including PANSS Negative Score interactions, reference group = SZAVH-, reference condition = Passive | | | | |  |
| --- | --- | --- | --- | --- | --- |
|  | Est | SE | df | t value | p-value |
| Fixed effects |  |  |  |  |  |
| Intercept | -5.53 | 0.90 | 116.86 | -6.15 | <.001 |
| Condition |  |  |  |  |  |
| Passive (reference) |  |  |  |  |  |
| Match | -0.27 | 0.44 | 198.00 | -0.61 | 0.540 |
| Mismatch | 0.82 | 0.44 | 198.00 | 1.86 | 0.065 |
| Group |  |  |  |  |  |
| SZAVH- (reference) |  |  |  |  |  |
| SZAVH+ | 1.89 | 0.87 | 137.63 | 2.18 | 0.031 |
| Location |  |  |  |  |  |
| Australia |  |  |  |  |  |
| Hong Kong | 1.27 | 0.86 | 99.00 | 1.47 | 0.143 |
| PANSS Negative Score | -0.10 | 0.08 | 131.72 | -1.30 | 0.196 |
| Condition x Group |  |  |  |  |  |
| Passive x SZAVH- (reference) |  |  |  |  |  |
| Match x SZAVH+ | -0.99 | 0.60 | 198.00 | -1.65 | 0.101 |
| Mismatch x SZAVH+ | -1.49 | 0.60 | 198.00 | -2.49 | 0.014 |
| PANSS Negative x Group |  |  |  |  |  |
| PANSS Negative x SZAVH - (reference) |  |  |  |  |  |
| PANSS Negative x SZAVH + | 0.19 | 0.13 | 138.25 | 1.51 | 0.134 |
| PANSS Negative x Condition |  |  |  |  |  |
| PANSS Negative x Passive (reference) |  |  |  |  |  |
| PANSS Negative x Match | 0.01 | 0.05 | 198.00 | 0.09 | 0.930 |
| PANSS Negative x Mismatch | 0.06 | 0.05 | 198.00 | 1.08 | 0.282 |
| PANSS Negative x Group x Condition |  |  |  |  |  |
| PANSS Negative x SZAVH - x Passive (reference) |  |  |  |  |  |
| PANSS Negative x SZAVH + x Match | 0.02 | 0.09 | 198.00 | 0.19 | 0.847 |
| PANSS Negative x SZAVH + Mismatch | -0.14 | 0.09 | 198.00 | -0.161 | 0.109 |
|  |  |  |  |  |  |
| Random effects: |  |  |  |  |  |
| Groups | Name | Variance | SD |  |  |
| Participants | Intercept | 12.41 | 3.52 |  |  |
| Residual |  | 4.05 | 2.01 |  |  |
| Number of observations: 297, participants: 99 | | | |  |  |

*PANSS General Score.* The inclusion of this variable in the model did not improve model fit (Baseline model: AIC = 1512, BIC = 1545, LL = -747.0, Model with covariate: AIC = 1514, BIC = 1550, LL = -746.9, Chi square difference (1) = 0.23, *p*= 0.630, and the *Group* x *Condition* interaction retained significance: F(2,198) = 3.34, *p*=0.037. Consistent with this, PANSS general score was not a significant predictor of N1 amplitude F(1,99) = 0.23, p=0.630, Est = -0.02, SE = 0.05, t(99) = -0.48, *p*=0.631, see Supplementary Table M. Further, when included in the model, the interaction between *PANSS General x Condition x Group* was not statistically significant: F(2, 198) = 0.06, *p*=0.940, see Supplementary Table N.

| **Supplementary Table M.** Linear Mixed Effects Model for N1 in clinical groups, including PANSS General Score, reference group = SZAVH-, reference condition = Passive | | | | |  |
| --- | --- | --- | --- | --- | --- |
|  | Est | SE | df | t value | p-value |
| Fixed effects |  |  |  |  |  |
| Intercept | -5.86 | 0.84 | 118.88 | -7.01 | <.001 |
| Condition |  |  |  |  |  |
| Passive (reference) |  |  |  |  |  |
| Match | -0.26 | 0.43 | 198.00 | -0.61 | 0.545 |
| Mismatch | 0.95 | 0.43 | 198.00 | 2.18 | 0.030 |
| Group |  |  |  |  |  |
| SZAVH- (reference) |  |  |  |  |  |
| SZAVH+ | 1.97 | 0.83 | 138.94 | 2.38 | 0.018 |
| Location |  |  |  |  |  |
| Australia |  |  |  |  |  |
| Hong Kong | 1.43 | 0.85 | 99.00 | 1.68 | 0.097 |
| PANSS General Score | 0.02 | 0.05 | 99.00 | -0.48 | 0.631 |
| Condition x Group |  |  |  |  |  |
| Passive x SZAVH- (reference) |  |  |  |  |  |
| Match x SZAVH+ | -1.04 | 0.58 | 198.00 | -1.78 | 0.077 |
| Mismatch x SZAVH+ | -1.46 | 0.58 | 198.00 | -2.52 | 0.013 |
|  |  |  |  |  |  |
| Random effects: |  |  |  |  |  |
| Groups | Name | Variance | SD |  |  |
| Participants | Intercept | 12.61 | 3.55 |  |  |
| Residual |  | 4.14 | 2.03 |  |  |
| Number of observations: 297, participants: 99 | | | |  |  |

| **Supplementary Table N.** Linear Mixed Effects Model for N1 in clinical groups, including PANSS General Score interactions, reference group = SZAVH-, reference condition = Passive | | | | |  |
| --- | --- | --- | --- | --- | --- |
|  | Est | SE | df | t value | p-value |
| Fixed effects |  |  |  |  |  |
| Intercept | -5.69 | 0.82 | 119.12 | -6.87 | <.001 |
| Condition |  |  |  |  |  |
| Passive (reference) |  |  |  |  |  |
| Match | -0.27 | 0.43 | 198.00 | -0.62 | 0.537 |
| Mismatch | 0.94 | 0.43 | 198.00 | 2.18 | 0.031 |
| Group |  |  |  |  |  |
| SZAVH- (reference) |  |  |  |  |  |
| SZAVH+ | 1.96 | 0.82 | 139.96 | 2.39 | 0.018 |
| Location |  |  |  |  |  |
| Australia |  |  |  |  |  |
| Hong Kong | 1.19 | 0.85 | 99.00 | 1.40 | 0.165 |
| PANSS General Score | -0.13 | 0.07 | 133.36 | -1.82 | 0.071 |
| Condition x Group |  |  |  |  |  |
| Passive x SZAVH- (reference) |  |  |  |  |  |
| Match x SZAVH+ | -1.03 | 0.58 | 198.00 | -1.78 | 0.077 |
| Mismatch x SZAVH+ | -1.46 | 0.58 | 198.00 | -2.52 | 0.013 |
| PANSS Negative x Group |  |  |  |  |  |
| PANSS General x SZAVH - (reference) |  |  |  |  |  |
| PANSS General x SZAVH + | 0.19 | 0.10 | 139.18 | 1.94 | 0.055 |
| PANSS General x Condition |  |  |  |  |  |
| PANSS General x Passive (reference) |  |  |  |  |  |
| PANSS General x Match | 0.04 | 0.05 | 198.00 | 0.79 | 0.432 |
| PANSS General x Mismatch | 0.04 | 0.04 | 198.00 | 0.89 | 0.372 |
| PANSS General x Group x Condition |  |  |  |  |  |
| PANSS General x SZAVH - x Passive (reference) |  |  |  |  |  |
| PANSS General x SZAVH + x Match | -0.02 | 0.07 | 198.00 | -0.26 | 0.780 |
| PANSS General x SZAVH + Mismatch | -0.06 | 0.07 | 198.00 | -0.914 | 0.362 |
|  |  |  |  |  |  |
| Random effects: |  |  |  |  |  |
| Groups | Name | Variance | SD |  |  |
| Participants | Intercept | 12.17 | 3.49 |  |  |
| Residual |  | 4.10 | 2.03 |  |  |
| Number of observations: 297, participants: 99 | | | |  |  |

*PSYRATS Delusion Score.* The inclusion of this variable in the model did not improve model fit (Baseline model: AIC = 1446, BIC = 1479, LL = -714.2, Model with covariate: AIC = 1447, BIC = 1484, LL = -713.7, Chi square difference (1) = 0.95, *p*= 0.330, and the *Group x Condition* interaction retained significance: F(2,198) = 3.34, *p*=0.037. Consistent with this, PSYRATS delusion score was not a significant predictor of N1 amplitude F (1, 95) = 0.96, p=0.331, Est = 0.05, SE = 0.05, t(95) = 0.98, *p*=0.331, see Supplementary Table O. Further, when included in the model, the interaction between *PSYRATS Delusion score x Condition x Group* was not statistically significant: F(2, 190) = 0.24, *p*=0.791, see Supplementary Table P.

| **Supplementary Table O.** Linear Mixed Effects Model for N1 in clinical groups, including PSYRATS Delusion Score, reference group = SZAVH-, reference condition = Passive | | | | |  |
| --- | --- | --- | --- | --- | --- |
|  | Est | SE | df | t value | p-value |
| Fixed effects |  |  |  |  |  |
| Intercept | -6.51 | 0.87 | 113.56 | -7.49 | <.001 |
| Condition |  |  |  |  |  |
| Passive (reference) |  |  |  |  |  |
| Match | -0.34 | 0.44 | 190.00 | -0.77 | 0.440 |
| Mismatch | 0.90 | 0.44 | 190.00 | 2.03 | 0.044 |
| Group |  |  |  |  |  |
| SZAVH- (reference) |  |  |  |  |  |
| SZAVH+ | 1.63 | 0.89 | 129.43 | 1.83 | 0.070 |
| Location |  |  |  |  |  |
| Australia |  |  |  |  |  |
| Hong Kong | 1.93 | 0.78 | 95.00 | 2.48 | 0.015 |
| PSYRATS Delusion Score | 0.05 | 0.05 | 95.00 | -1.70 | 0.091 |
| Condition x Group |  |  |  |  |  |
| Passive x SZAVH- (reference) |  |  |  |  |  |
| Match x SZAVH+ | -1.01 | 0.60 | 190.00 | -1.70 | 0.091 |
| Mismatch x SZAVH+ | -1.51 | 0.60 | 190.00 | -2.54 | 0.012 |
|  |  |  |  |  |  |
| Random effects: |  |  |  |  |  |
| Groups | Name | Variance | SD |  |  |
| Participants | Intercept | 11.56 | 3.40 |  |  |
| Residual |  | 4.16 | 2.04 |  |  |
| Number of observations: 285, participants: 95 | | | |  |  |

| **Supplementary Table P.** Linear Mixed Effects Model for N1 in clinical groups, including PSYRATS Delusion Score interactions, reference group = SZAVH-, reference condition = Passive | | | | |  |
| --- | --- | --- | --- | --- | --- |
|  | Est | SE | df | t value | p-value |
| Fixed effects |  |  |  |  |  |
| Intercept | -6.86 | 0.93 | 121.54 | -7.38 | <.001 |
| Condition |  |  |  |  |  |
| Passive (reference) | - | - | - | - | - |
| Match | -0.45 | 0.56 | 190.00 | -0.80 | 0.424 |
| Mismatch | 1.23 | 0.56 | 190.00 | 2.21 | 0.029 |
| Group |  |  |  |  |  |
| SZAVH- (reference) | - | - | - | - | - |
| SZAVH+ | 2.28 | 1.32 | 136.82 | 1.73 | 0.086 |
| Location |  |  |  |  |  |
| Australia |  |  |  |  |  |
| Hong Kong | 1.88 | 0.78 | 95.00 | 2.42 | 0.018 |
| PSYRATS Delusion Score | 0.12 | 0.09 | 136.81 | 1.42 | 0.159 |
| Condition x Group |  |  |  |  |  |
| Passive x SZAVH- (reference) |  |  |  |  |  |
| Match x SZAVH+ | -0.64 | 0.96 | 190.00 | -0.67 | 0.507 |
| Mismatch x SZAVH+ | -1.29 | 0.96 | 190.00 | -1.34 | 0.181 |
| PSYRATS Delusion Score x Group |  |  |  |  |  |
| PSYRATS Delusion Score x SZAVH - (reference) |  |  |  |  |  |
| PSYRATS Delusion Score x SZAVH + | -0.09 | 0.12 | 136.65 | -0.81 | 0.419 |
| PANSS Negative x Condition |  |  |  |  |  |
| PSYRATS Delusion Score x Passive (reference) |  |  |  |  |  |
| PSYRATS Delusion Score x Match | 0.02 | 0.06 | 190.00 | 0.30 | 0.764 |
| PSYRATS Delusion Score x Mismatch | -0.06 | 0.06 | 190.00 | -0.96 | 0.339 |
| PSYRATS Delusion Score x Group x Condition |  |  |  |  |  |
| PSYRATS Delusion Score x SZAVH - x Passive (reference) |  |  |  |  |  |
| PSYRATS Delusion Score x SZAVH + x Match | -0.04 | 0.08 | 190.00 | -0.49 | 0.622 |
| PSYRATS Delusion Score x SZAVH + Mismatch | 0.01 | 0.08 | 190.00 | 0.166 | 0.869 |
|  |  |  |  |  |  |
| Random effects: |  |  |  |  |  |
| Groups | Name | Variance | SD |  |  |
| Participants | Intercept | 11.45 | 3.38 |  |  |
| Residual |  | 4.11 | 2.03 |  |  |
| Number of observations: 285, participants: 95 | | | |  |  |

*Olanzapine-Equivalent Medication Dosage.* The inclusion of this variable in the model did not improve model fit (Baseline model: AIC = 1176, BIC = 1208, LL = -579.0, Model with covariate: AIC = 1178, BIC = 1213, LL = -579.0, Chi square difference (1) = 0.13, *p*=0.721. The *Group x Condition* interaction became non-significant: F(2,164) = 1.87, *p*=0.158. The olanzapine dosage score was not a significant predictor of N1 amplitude F (1, 82) = 0.13, p=0.722, Est = 0.01, SE = 0.03, t(82) = 0.36, *p*=0.722, see Supplementary Table Q. Further, when included in the model, the interaction between *Olanzapine dosage score x Condition x Group* was not statistically significant: F(2, 164) = 0.44, *p*=0.643, Est = -0.04, SE = 0.05, t(164) = -0.69, *p*=0.492, see Supplementary Table R. The non-significant *Group x Condition* interaction can likely be attributed to a reduction in power due to a smaller number of participants providing data on medication dosage (n = 82), given the negligible association between Olanzapine dosage and N1 amplitude in this analysis.

| **Supplementary Table Q.** Linear Mixed Effects Model for N1 in clinical groups, including Olanzapine Equivalent Dosage, reference group = SZAVH-, reference condition = Passive | | | | |  |
| --- | --- | --- | --- | --- | --- |
|  | Est | SE | df | t value | p-value |
| Fixed effects |  |  |  |  |  |
| Intercept | -4.35 | 0.71 | 103.76 | -6.11 | <.001 |
| Condition |  |  |  |  |  |
| Passive (reference) |  |  |  |  |  |
| Match | -0.31 | 0.42 | 164.00 | -0.75 | 0.458 |
| Mismatch | 0.61 | 0.42 | 164.00 | 1.46 | 0.147 |
| Group |  |  |  |  |  |
| SZAVH- (reference) |  |  |  |  |  |
| SZAVH+ | 1.23 | 0.75 | 121.39 | 1.64 | 0.103 |
| Location |  |  |  |  |  |
| Australia |  |  |  |  |  |
| Hong Kong | 0.51 | 0.72 | 82.00 | 0.72 | 0.474 |
| Olanzapine Equivalent Dosage | 0.01 | 0.03 | 82.00 | 0.36 | 0.722 |
| Condition x Group |  |  |  |  |  |
| Passive x SZAVH- (reference) |  |  |  |  |  |
| Match x SZAVH+ | -0.53 | 0.56 | 164.00 | -0.94 | 0.347 |
| Mismatch x SZAVH+ | -1.09 | 0.56 | 164.00 | -1.93 | 0.055 |
|  |  |  |  |  |  |
| Random effects: |  |  |  |  |  |
| Groups | Name | Variance | SD |  |  |
| Participants | Intercept | 7.58 | 2.75 |  |  |
| Residual |  | 3.24 | 1.80 |  |  |
| Number of observations: 246, participants: 82 | | | |  |  |

| **Supplementary Table R.** Linear Mixed Effects Model for N1 in clinical groups, including PSYRATS Delusion Score interactions, reference group = SZAVH-, reference condition = Passive | | | | |  |
| --- | --- | --- | --- | --- | --- |
|  | Est | SE | df | t value | p-value |
| Fixed effects |  |  |  |  |  |
| Intercept | -4.24 | 0.71 | 105.91 | -5.95 | <.001 |
| Condition |  |  |  |  |  |
| Passive (reference) |  |  |  |  |  |
| Match | -0.26 | 0.43 | 164.00 | -0.60 | 0.549 |
| Mismatch | 0.66 | 0.43 | 164.00 | 1.51 | 0.132 |
| Group |  |  |  |  |  |
| SZAVH- (reference) |  |  |  |  |  |
| SZAVH+ | 1.18 | 0.75 | 124.80 | 1.58 | 0.116 |
| Location |  |  |  |  |  |
| Australia |  |  |  |  |  |
| Hong Kong | 0.56 | 0.70 | 82.01 | 0.81 | 0.420 |
| Olanzapine Equivalent Dosage | 0.06 | 0.05 | 124.67 | 1.15 | 0.252 |
| Condition x Group |  |  |  |  |  |
| Passive x SZAVH- (reference) |  |  |  |  |  |
| Match x SZAVH+ | -0.65 | 0.59 | 164.00 | -1.13 | 0.262 |
| Mismatch x SZAVH+ | -1.09 | 0.58 | 164.00 | -1.87 | 0.064 |
| Olanzapine Equivalent Dosage x Group |  |  |  |  |  |
| Olanzapine Equivalent Dosage x SZAVH - (reference) |  |  |  |  |  |
| Olanzapine Equivalent Dosage x SZAVH + | -0.09 | 0.07 | 124.81 | -1.36 | 0.176 |
| PANSS Negative x Condition |  |  |  |  |  |
| Olanzapine Equivalent Dosage x Passive (reference) |  |  |  |  |  |
| Olanzapine Equivalent Dosage x Match | 0.02 | 0.05 | 164.00 | 0.40 | 0.689 |
| Olanzapine Equivalent Dosage x Mismatch | 0.01 | 0.04 | 164.00 | 0.38 | 0.708 |
| Olanzapine Equivalent Dosage x Group x Condition |  |  |  |  |  |
| Olanzapine Equivalent Dosage x SZAVH - x Passive (reference) |  |  |  |  |  |
| Olanzapine Equivalent Dosage x SZAVH + x Match | 0.01 | 0.05 | 164.00 | 0.21 | 0.833 |
| Olanzapine Equivalent Dosage x SZAVH + Mismatch | -0.04 | 0.05 | 164.00 | -0.69 | 0.492 |
|  |  |  |  |  |  |
| Random effects: |  |  |  |  |  |
| Groups | Name | Variance | SD |  |  |
| Participants | Intercept | 7.62 | 2.71 |  |  |
| Residual |  | 3.19 | 1.79 |  |  |
| Number of observations: 246, participants: 82 | | | |  |  |

*Association between difference in N1 amplitude in Match and Passive Conditions and Clinical Variables.*

A multiple linear regression analysis predicting difference in N1 amplitude in the match and passive conditions was not statistically significant F(4,90) = 0.56, *p*=0.692, explaining 2.4% of the variance in the N1 amplitude difference score. No individual predictor reached significance: PANSS Negative Symptoms (B = -0.01, SE = 0.08, *t* = 0.05, *p* = 0.961), PANSS Positive Symptoms (B = -0.05, SE = 0.07, *t* = -0.67, *p*=0.504), PANSS General Symptoms (B = 0.08, SE = 0.07, t = 1.12, p = 0.266), PSYRATS Delusion score (B = -0.06, SE = 0.06, *t* = -0.96, *p* = 0.33). Univariate investigations of each of these variables and their association with the N1 amplitude difference score revealed no significant associations: PANSS Negative Symptoms (r = 0.03, *p* = 0.483), PANSS Positive Symptoms (r = -0.01, *p*=0.898), PANSS General Symptoms (r = 0.03, *p*=0.415), PSYRATS Delusion score (r = -0.04, *p =* 0.434).

**Spearman Rank-Order correlations between N1 difference scores (Match minus Passive, Match minus Mismatch, and Passive minus Mismatch) and the clinical participants scores on the clinical variables (namely, PANSS Positive Total, PANSS Negative Total, PANSS General Total, PSYRATS Auditory Hallucinations, PSYRATS Delusions, Olanzapine-Equivalent Medication Dosage, Age, and Sex)**

| Variable | Spearman Rank-Order (rho) | N1: Match minus Passive | N1: Match minus Mismatch | N1: Mismatch minus Passive |
| --- | --- | --- | --- | --- |
| N1: Match minus Passive | Correlation Coefficient | 1.000 | .477^**^ | .557^**^ |
|  | Sig. (2-tailed) | . | <.001 | <.001 |
|  | N | 142 | 142 | 142 |
| N1: Match minus Mismatch | Correlation Coefficient | .477^**^ | 1.000 | -.333^**^ |
|  | Sig. (2-tailed) | <.001 | . | <.001 |
|  | N | 142 | 142 | 142 |
| N1: Mismatch minus Passive | Correlation Coefficient | .557^**^ | -.333^**^ | 1.000 |
|  | Sig. (2-tailed) | <.001 | <.001 | . |
|  | N | 142 | 142 | 142 |
| PANSS Positive Total | Correlation Coefficient | -.041 | .040 | -.112 |
|  | Sig. (2-tailed) | .684 | .691 | .270 |
|  | N | 99 | 99 | 99 |
| PANSS Negative Total | Correlation Coefficient | .013 | -.052 | .078 |
|  | Sig. (2-tailed) | .901 | .610 | .445 |
|  | N | 99 | 99 | 99 |
| PANSS General Total | Correlation Coefficient | .016 | -.059 | .043 |
|  | Sig. (2-tailed) | .878 | .560 | .671 |
|  | N | 99 | 99 | 99 |
| PSYRATS Auditory Hallucinations | Correlation Coefficient | -.222^*^ | .128 | -.400^**^ |
|  | Sig. (2-tailed) | .027 | .207 | <.001 |
|  | N | 99 | 99 | 99 |
| PSYRATS Delusions | Correlation Coefficient | -.117 | .074 | -.198 |
|  | Sig. (2-tailed) | .260 | .478 | .054 |
|  | N | 95 | 95 | 95 |
| Olanzapine Equivalent Dosage | Correlation Coefficient | .061 | .035 | -.063 |
|  | Sig. (2-tailed) | .584 | .756 | .572 |
|  | N | 82 | 82 | 82 |
| Age | Correlation Coefficient | -.022 | -.080 | -.002 |
|  | Sig. (2-tailed) | .791 | .346 | .985 |
|  | N | 142 | 142 | 142 |
| Sex | Correlation Coefficient | .015 | .010 | .094 |
|  | Sig. (2-tailed) | .863 | .901 | .267 |
|  | N | 142 | 142 | 142 |
